# Retrospective analysis of transarterial chemoembolization or hepatic arterial infusion chemotherapy combined with lenvatinib with or without PD-1 inhibitor as first-line therapy for unresectable hepatocellular carcinoma with high tumor burden: a propensity score-matched study

**DOI:** 10.3389/fimmu.2026.1717797

**Published:** 2026-02-16

**Authors:** Zhenfeng Li, Huiyong Wu, Ran Xu, Xu Chang, Shushan Wang, Peng Sun

**Affiliations:** 1 Department of Intervention Oncology, Shandong Cancer Hospital and Institute, Shandong First Medical University and Shandong Academy of Medical Sciences, Jinan, Shandong, China; 2 Department of Medical Imaging Department, Shandong Cancer Hospital and Institute, Shandong First Medical University and Shandong Academy of Medical Sciences, Jinan, Shandong, China; 3 Department of Intervention Oncology, Tai’an Cancer Hospital, Tai’an, Shandong, China

**Keywords:** hepatic arterial infusion chemotherapy, high tumor burden, lenvatinib, liver function, programmed death 1 inhibitors, transarterial chemoembolization, unresectable hepatocellular carcinoma

## Abstract

**Purpose:**

To compare the efficacy, safety, and hepatic impact of TACE or HAIC plus lenvatinib with or without PD-1 inhibitors in unresectable hepatocellular carcinoma with high tumor burden (HTB-uHCC).

**Patients and methods:**

This retrospective study (2019–2023) included 278 HTB-uHCC patients (defined as tumors exceeding the up-to-11 criteria or exhibiting Vp4 portal vein tumor thrombus) receiving either doublet therapy (TACE or HAIC + lenvatinib, THL) or triplet therapy (TACE or HAIC + lenvatinib + PD-1 inhibitor, THLP) (139 per cohort after 1:1 propensity score matching; caliper=0.2). Primary endpoints included overall survival (OS) and progression-free survival (PFS). Secondary endpoints comprised objective response rate (ORR), serial liver function tests, and adverse events (AEs).

**Results:**

The THLP group demonstrated superior OS (median22.4 vs. 17.6 months, HR = 0.55, P < 0.001), PFS (13.5 vs. 8.5 months, HR = 0.53, P < 0.001), and ORR (72.7% vs. 52.5%, P < 0.001) compared to the THL group. No intergroup differences in albumin-bilirubin (ALBI) scores were observed at baseline or during months 1–5 (all P > 0.05). Both cohorts showed significant ALBI deterioration at progression versus baseline (P < 0.001). Child-Pugh stability at 6 months independently predicted ORR (adjusted OR 8.71, 95% CI 4.93–15.40; P <0.001). Grade 3–4 AEs occurred at comparable rates (46.8%vs. 43.2%,P=0.629), with no treatment-related deaths.

**Conclusion:**

Triplet therapy significantly improves survival and tumor response without accelerating early liver function decline in patients with HTB-uHCC. Child-Pugh stability correlates strongly with treatment efficacy.

## 1 Introduction

Hepatocellular carcinoma (HCC) remains a global health challenge, ranking as the third leading cause of cancer-related mortality (1, 2). Surgical resection offers curative potential for early-stage hepatocellular carcinoma (HCC). However, in China, more than 80% of patients are diagnosed with unresectable HCC (uHCC) at initial presentation. A substantial proportion of these uHCC cases are associated with a high tumor burden, which is commonly defined as a tumor burden exceeding the up-to-11 criteria or the presence of Vp4 portal vein tumor thrombus (PVTT) (3–5). These subgroups face a dismal prognosis, with median overall survival (OS) of 2.7–7.6 months under conventional therapies (6, 7), underscoring an urgent need for effective first-line strategies. While systemic therapy remains the cornerstone for advanced uHCC according to NCCN guidelines (8), first-line options have diversified to include immune checkpoint inhibitor (ICI) combinations such as atezolizumab-bevacizumab, dual ICIs or tyrosine-kinase inhibitors (TKIs) monotherapy (such as Sorafenib or lenvatinib) (9–11). In China, lenvatinib combined with Programmed Death 1(PD-1) inhibitors has emerged as the most prevalent first-line regimen due to its accessibility under national reimbursement policies. This preference is supported by the REFLECT trial, in which lenvatinib demonstrated non-inferior OS compared to sorafenib and higher objective response rates (12). The subsequent LEAP-002 trial further validated this strategy, showing a trend toward improved OS with lenvatinib-pembrolizumab versus lenvatinib monotherapy, though statistical significance was not achieved (13). However, systemic therapy alone yields suboptimal outcomes for uHCC with HTB (HTB-uHCC). As reported in the IMbrave150 update, patients with HTB (Vp4 PVTT or tumor occupancy ≥50%) achieved a median OS of only 7.6 months and progression-free survival (PFS) of 5.4 months with atezolizumab-bevacizumab (6). This suboptimal outcome is primarily due to accelerated hepatic decompensation caused by tumor progression (14). In response, Chinese clinical guidelines recommend integrating locoregional therapies (such as transarterial chemoembolization [TACE] or hepatic arterial infusion chemotherapy [HAIC]) with systemic treatment to achieve rapid cytoreduction. (9, 15). Despite this advanced treatment strategy, the specific benefit of adding a PD-1 inhibitor to the combination of TACE/HAIC and lenvatinib (forming a triplet regimen) in patients with high-tumor-burden unresectable hepatocellular carcinoma (HTB-uHCC) remains unclear. This is because existing studies often include mixed patient populations.. Therefore, this study was designed to address this gap. We conducted a propensity score-matched analysis to directly compare the efficacy and safety of doublet therapy (TACE or HAIC plus lenvatinib) versus triplet therapy (TACE or HAIC plus lenvatinib and a PD-1 inhibitor) as a first-line treatment for HTB-uHCC. By precisely evaluating survival benefits, liver function changes, and safety profiles within this high-risk cohort, our findings aim to provide evidence for personalized treatment strategies and inform the design of future randomized controlled trials.

## 2 Materials and methods

### 2.1 Study design and patients

This retrospective, PSM study analyzed patients with HTB-uHCC who received first-line therapy with TACE or HAIC, lenvatinib, and/or PD-1 inhibitors. The inclusion criteria were as follows: 1) Histologically or clinically confirmed uHCC per AASLD/EASL guidelines with HTB, defined as: a) Tumors exceeding the up-to-11 criteria (sum of tumor number and largest diameter >11); b) Portal vein tumor thrombus (PVTT) classified as Vp4 (involvement of main portal vein trunk), with specific clarification: All included Vp4 cases exhibited partial portal vein occlusion (<70% luminal obstruction) confirmed by triphasic CT/MRI. Complete portal vein occlusion (≥70% obstruction) was considered a contraindication for TACE per institutional protocol and thus excluded; 2) No prior systemic or locoregional therapies; 3) Aged 18 to 75 years; 4) ECOG performance status 0–1;; 4) Child-Pugh class A or B7 liver function; 5) Adequate hematologic and organ function, defined as: a)Coagulation:​ International normalized ratio (INR) ≤ 1.5; b)Hematologic:​ Platelet count ≥ 50 × 10^9^/L; hemoglobin ≥ 8.5 g/dL; absolute neutrophil count ≥ 1.5 × 10^9^/L; c)Renal:​ Serum creatinine ≤ 1.5 × the upper limit of normal (ULN); 6) Received ≥2 cycles of TACE or HAIC combined with Lenvatinib ± PD-1 inhibitors. The exclusion criteria were as follows:1) Prior liver transplantation or concurrent malignancies;2) Severe cardiovascular, renal, or immune-related comorbidities.

### 2.2 Treatment procedures

All enrolled patients underwent evaluation by a Multi-Disciplinary Team (MDT) at Shandong First Medical University Affiliated Cancer Hospital, which unanimously recommended first-line systemic therapy combined with TACE or HAIC for HTB-uHCC. The decision to add PD-1 inhibitors was made collaboratively by the treating physicians, patients, and their families, considering factors such as treatment affordability, performance status, and liver function. **Supplementary Table S1** summarize the main reasons for non-use PD-1 inhibitors in the THL group.

#### 2.2.1 Trans-arterial therapy

The choice between TACE and HAIC followed standardized institutional criteria established by the Multidisciplinary Hepatobiliary Tumor Board. Specific clinical and technical indications for each modality are detailed in **Supplementary Table S2**.

#### 2.2.2 Transarterial chemoembolization

The patient is routinely placed in the supine position. The puncture site is disinfected, draped, and locally infiltrated with anesthetic. The Seldinger technique is used for percutaneous puncture of the femoral artery (or the radial artery if feasible), followed by placement of a 5-French catheter sheath. Conventional angiography of the celiac artery or common hepatic artery is performed, with image acquisition covering the arterial, parenchymal, and venous phases. If areas of sparse/absent vascularity or incomplete tumor staining are observed, angiography of the superior mesenteric artery, left gastric artery, inferior phrenic artery, renal artery, internal thoracic artery, intercostal artery, or lumbar artery is performed to identify ectopic hepatic arteries or extrahepatic collateral feeding vessels. CBCT was routinely employed for all TACE procedures regardless of tumor-feeding artery visibility on digital subtraction angiography (DSA). A microcatheter (Progreat, Terumo Interventional Systems) is then superselectively advanced into the tumor-feeding arterial branches. After confirming tumor staining via angiography, embolization is performed using a chemotherapeutic lipiodol emulsion. The embolization endpoint is defined as dense lipiodol deposition in the tumor area and visualization of small portal vein branches around the tumor. Subsequently, absorbable gelatin sponge particles or Embosphere microspheres of appropriate size are injected to achieve complete blood flow stasis, with the endpoint characterized by a dry branch appearance of the feeding artery. After sheath removal, manual compression is applied proximal to the femoral puncture site for 5–15 minutes, followed by pressure dressing, ensuring palpable dorsalis pedis artery pulsation on the punctured side. The patient is advised to remain in bed with the punctured limb immobilized for ≥6 hours, and the bandage is removed after 12–24 hours. Common chemotherapeutic agents for TACE include: 1) Anthracyclines (e.g., doxorubicin, epirubicin), 2)-Platinum agents (e.g., oxaliplatin, cisplatin, lobaplatin), and- 3) Mitomycin, fluorouracil, raltitrexed, or hydroxycamptothecin. Single-agent regimens typically use anthracyclines or platinum agents, while combination regimens may include 2–3 agents. The choice and dosage depend on tumor burden, body surface area, liver and kidney function, blood cell counts, performance status, and comorbidities.

#### 2.2.3 Hepatic artery infusion chemotherapy

The procedural steps for HAIC align with those described in our team’s previously published articles and are similar to conventional TACE procedures. HAIC typically employs the Seldinger technique, involving percutaneous puncture of the femoral artery (or alternative access sites such as the radial or subclavian arteries) for catheter placement. The catheter is advanced to perform angiography of the celiac trunk and superior mesenteric artery. Special attention is given to identifying tumor collateral feeding arteries; additional angiography of the phrenic artery, intercostal artery, right renal artery, or right internal thoracic artery may be performed if necessary to comprehensively map the tumor’s vascular supply. Based on the tumor’s vascular anatomy, the catheter is superselectively positioned in the dominant tumor-feeding artery. If the tumor receives dual blood supply from both the celiac trunk and superior mesenteric artery or has additional feeding vessels, non-dominant branches may be embolized before placing the catheter in the primary feeding artery. Alternatively, sequential infusions may be administered through different feeding arteries. After catheter placement, 5–10 mL of heparinized saline (10,000 units of heparin diluted in 100 mL of 0.9% sodium chloride solution at a ratio of 100:1) is bolus-injected to prevent catheter occlusion. The external segment of the catheter is covered with sterile gauze and secured to the surrounding skin using a transparent adhesive dressing. The patient remains supine for continuous intra-arterial chemotherapy infusion, with the catheterized limb immobilized to avoid bending or exertion, which could dislodge the catheter. Upon completion of the infusion, the sheath and catheter are removed, and the puncture site is compressed and bandaged. The HAIC chemotherapy regimen follows the FOLFOX protocol, detailed as follows: - Oxaliplatin: 85 mg/m² or 130 mg/m², administered via intra-arterial infusion over 2–3 hours. - Leucovorin (Calcium Folinate): 400 mg/m² or Levoleucovorin: 200 mg/m², delivered via intra-arterial infusion over 1–2 hours. - 5-Fluorouracil (5-FU): - Bolus injection: 400 mg/m² via intra-arterial route. - Continuous infusion: 2400 mg/m² via intra-arterial route over 23 or 46 hours. Each cycle was scheduled at 4-week intervals. The number of cycles administered was determined by treatment response and the physician’s discretion.

### 2.3 Data collection, follow-up, and assessments

This retrospective study extracted data from electronic medical records (EMR) and the Picture Archiving and Communication System (PACS), with protocol-mandated evaluations conducted every 4 weeks ( ± 3 days) during the first year and every 6–12 weeks thereafter until progression or death. Each visit encompassed triple-phase contrast-enhanced CT or MRI (slice thickness ≤3-4mm) interpreted by two blinded radiologists. Tumor response was evaluated using the modified Response Evaluation Criteria in Solid Tumors (mRECIST). Tumor responses were categorized as follows: complete response (CR), partial response (PR), stable disease (SD), progressive disease (PD), objective response rate (ORR) and disease control rate (DCR). The ORR included CR and PR rates, while the DCR encompassed CR, PR, and SD. OS was calculated from treatment initiation to death from any cause; PFS encompassed time to radiologic progression or death. Laboratory assessments including liver function tests (albumin, bilirubin, for ALBI score calculation), complete blood count, coagulation profile, serum AFP quantification, and renal function markers; clinical documentation of ECOG performance status, Child-Pugh classification (ascites/encephalopathy grading), and targeted physical examination for regimen-specific toxicities. Adverse events (AEs) were systematically graded per CTCAE v3.0 with focused surveillance for lenvatinib-associated hypertension/proteinuria (weekly blood pressure logs, urine protein-creatinine ratio) and PD-1 inhibitor-related immune toxicities (TSH/fT4 monitoring q12w). Objective responses required confirmatory imaging ≥4 weeks after initial documentation, while progression triggered multidisciplinary review; all data underwent source verification against EMR with PACS archiving of imaging studies for centralized audit.

### 2.4 Statistical methodology

To mitigate baseline imbalances, we performed 1:1 greedy nearest-neighbor matching with a caliper of 0.2, implemented using the R package MatchIt. The following 17 clinically relevant covariates were included in the propensity score model based on their established prognostic significance in HCC: Sex, Age, ECOG performance status score, BMI, Child Pugh score at screening, Child Pugh class at screening, Albumin bilirubin grade at screening, HBsAg, Baseline α fetoprotein expression level, Largest tumor size, Tumor distribution, Number of tumors, Extrahepatic spread, Venous invasion, High tumor burden type, BCLC stage, and Transarterial therapies. After matching, we compared clinical outcomes between the two groups using appropriate statistical tests. Continuous variables were summarized as median (interquartile range, IQR) and analyzed using the Wilcoxon rank-sum test, while categorical variables were reported as frequencies and percentages and assessed via Fisher’s exact test (for expected frequencies <5) or the chi-squared test. Survival outcomes were evaluated using Kaplan-Meier curves with log-rank tests for inter-group comparisons. Multivariate Cox regression identified independent factors associated with OS and PFS. Within the THLP cohort, subgroup analyses employed multivariable Cox regression to determine OS predictors. Multivariate logistic regression was used to identified independent factors associated with ORR. All tests were two-sided, with p< 0.05 defining statistical significance. Analyses were conducted using R software (version 4.2.2).

## 3 Results

### 3.1 Baseline characteristics

**Figure 1:**
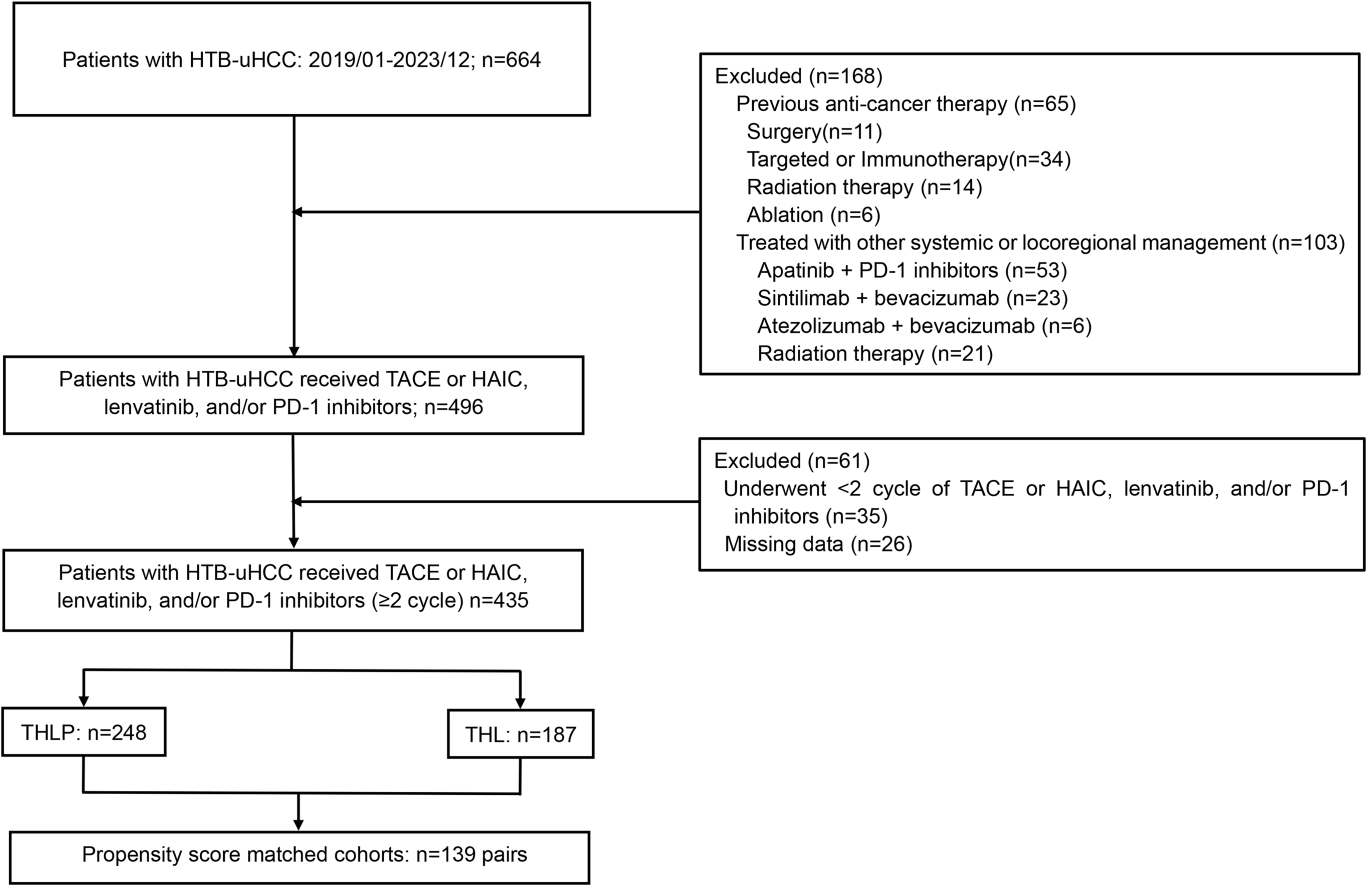
Flowchart of the patient selection process. HTB-uHCC, Unresectable Hepatocellular carcinoma with high tumor burden; THL, TACE Or HAIC combined with Lenvatinib; THLP, TACE Or HAIC combined with Lenvatinib and programmed death 1 inhibitors; HAIC, Hepatic Arterial Infusion Chemotherapy; TACE, Transarterial Chemoembolization.

A total of 664 patients with advanced HCC meeting the inclusion criteria were initially screened between January 2019 and December 2023, the median follow-up time for all patients was 31.2 months (range: 3.8–54.0 months). After applying the exclusion criteria (**Figure 1**), 435 patients were enrolled and divided into two groups. Following 1:1 PSM, 278 patients (139 per group) were included in the final analysis. **Table 1** provides a comprehensive comparison of baseline characteristics between the THLP and THL groups before and after PSM. After PSM, all covariates achieved balance (standardized mean differences < 0.1), confirming comparability (**Supplementary Figure S1**).

**Table 1:** Patient demographics and baseline characteristics before and after PSM.

Characteristics	Unmatched			Matched		
	THLP N = 248^1^	THL N = 187^1^	*P*-value^2^	THLP N = 139^1^	THL N = 139^1^	*P*-value^2^
Sex			0.179			0.733
Female	42 (17%)	23 (12%)		21 (15%)	19 (14%)	
Male	206 (83%)	164 (88%)		118 (85%)	120 (86%)	
Age (year)			0.109			0.411
Median (Q1, Q3)	56 (51, 62)	58 (52, 65)		56 (51, 61)	57 (52, 65)	
ECOG performance status score			0.019			0.694
0	97 (39%)	53 (28%)		40 (29%)	43 (31%)	
1	151 (61%)	134 (72%)		99 (71%)	96 (69%)	
BMI (kg/m²)			0.244			0.900
Median (Q1, Q3)	22.7 (20.8, 24.9)	23.0 (20.8, 25.4)		22.5 (20.8, 25.0)	22.7 (20.3, 25.4)	
Child Pugh score at screening			0.250			0.451
Median (Q1, Q3)	6.00 (5.00, 6.00)	6.00 (5.00, 6.00)		6.00 (5.00, 6.00)	6.00 (5.00, 6.00)	
Child Pugh class at screening			0.084			0.569
A	205 (83%)	142 (76%)		109 (78%)	105 (76%)	
B	43 (17%)	45 (24%)		30 (22%)	34 (24%)	
Albumin bilirubin grade at screening			0.896			0.903
1	101 (41%)	75 (40%)		56 (40%)	55 (40%)	
2	147 (59%)	112 (60%)		83 (60%)	84 (60%)	
Platelet count (X10^9^/L)			0.818			0.675
Median (Q1, Q3)	168 (124, 244)	170 (120, 249)		174 (126, 247)	169 (120, 259)	
Etiology of Liver disease			0.867			>0.999
HBV	200 (81%)	152 (81%)		114 (82%)	114 (82%)	
HCV	48 (19%)	35 (19%)		25 (18%)	25 (18%)	
Baseline α fetoprotein expression level (ng/mL)			0.059			0.890
Median (Q1, Q3)	2,767 (81, 44,510)	1,303 (29, 12,435)		2,112 (29, 19,500)	1,665 (29, 20,601)	
Largest tumor size (cm)			0.947			0.901
Median (Q1, Q3)	10.6 (7.6, 13.2)	10.5 (7.3, 13.6)		10.9 (7.8, 13.2)	10.6 (7.9, 13.8)	
Tumor distribution			0.079			0.807
Bilobar	133 (54%)	116 (62%)		81 (58%)	83 (60%)	
Unilobar	115 (46%)	71 (38%)		58 (42%)	56 (40%)	
Number of tumors			0.052			0.701
>3	160 (65%)	137 (73%)		92 (66%)	95 (68%)	
≤3	88 (35%)	50 (27%)		47 (34%)	44 (32%)	
Extrahepatic spread			0.419			0.896
No	185 (75%)	133 (71%)		97 (70%)	96 (69%)	
Yes	63 (25%)	54 (29%)		42 (30%)	43 (31%)	
Venous invasion			0.068			0.791
No	60 (24%)	60 (32%)		39 (28%)	41 (29%)	
Yes	188 (76%)	127 (68%)		100 (72%)	98 (71%)	
High tumor burden type			<0.001			0.899
Exceeded the up-to-11 criteria	113 (46%)	103 (55%)		71 (51%)	72 (52%)	
Exceeded the up-to-11 criteria And VP4 PVTT	36 (15%)	44 (24%)		31 (22%)	28 (20%)	
VP4 PVTT	99 (40%)	40 (21%)		37 (27%)	39 (28%)	
BCLC stage			0.008			0.647
B	34 (14%)	44 (24%)		25 (18%)	28 (20%)	
C	214 (86%)	143 (76%)		114 (82%)	111 (80%)	
Trans-arterial therapies			<0.001			0.317
HAIC	198 (80%)	98 (52%)		93 (67%)	85 (61%)	
TACE	50 (20%)	89 (48%)		46 (33%)	54 (39%)	

^1^n (%).

^2^Pearson's Chi-squared test; Wilcoxon rank sum test.

THL, TACE Or HAIC combined with Lenvatinib; THLP, TACE Or HAIC combined with Lenvatinib and programmed death 1 inhibitors.

PSM, propensity score matching; BMI, Body mass index; ECOG performance status score, Eastern Cooperative Oncology Group Performance Status.

HAIC, Hepatic Arterial Infusion Chemotherapy; TACE, Transarterial Chemoembolization; PVTT, Portal Vein Tumor Thrombus.

HBV, Hepatitis B Virus; HCV, Hepatitis C Virus;

### 3.2 Survival outcomes before and after PSM

**Figure 2:**
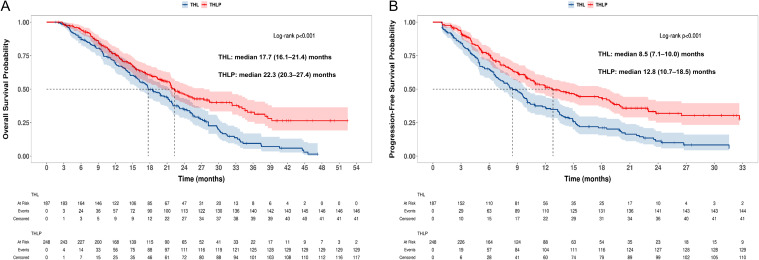
Kaplan-Meier curves of **(A)** overall survival and **(B)** progression-free survival before propensity score matching in two groups.

**Figure 3:**
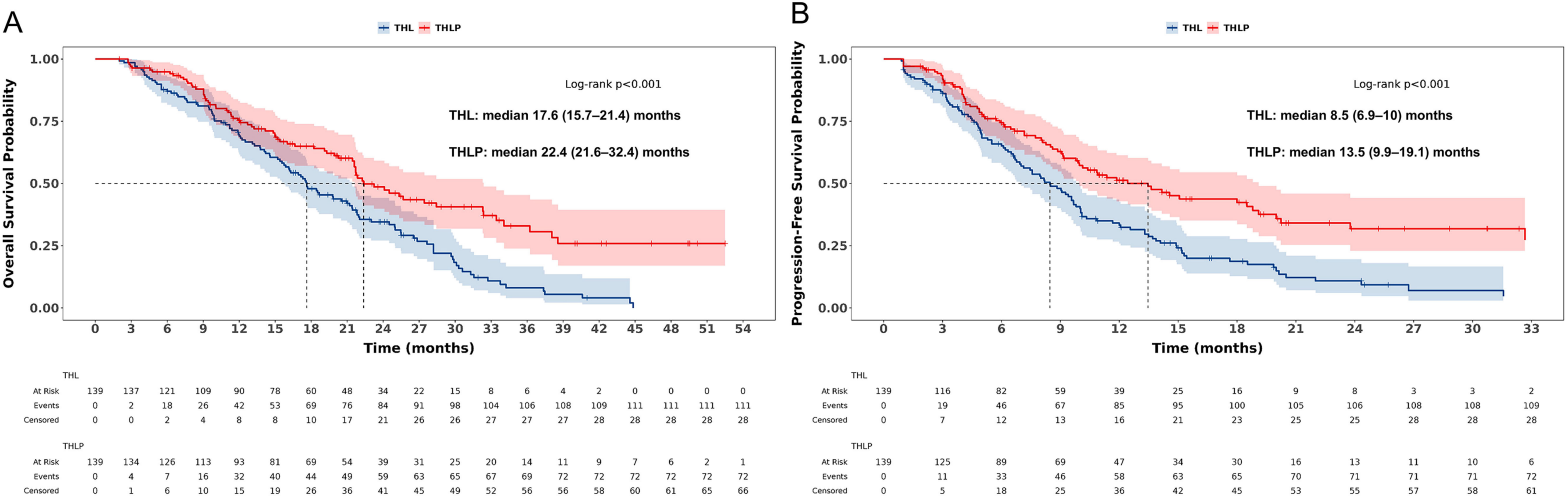
Kaplan-Meier curves of **(A)** overall survival and **(B)** progression-free survival after propensity score matching in two groups.

In both pre- and post-PSM analyses, the THLP group demonstrated significantly superior survival outcomes compared to the THL group. Before PSM, the median OS for the THLP group was 22.3 months versus 17.7 months for the THL group (HR = 0.60, 95% CI: 0.48–0.77, P< 0.001) (**Figure 2A**). PFS was 12.8 months vs. 8.5 months (HR = 0.55, 95% CI: 0.44–0.70, P < 0.001) (**Figure 2B**). After 1:1 matching, the survival advantage persisted, with a median OS of 22.4 months vs. 17.6months (HR = 0.55, 95% CI: 0.41–0.74, P < 0.001) (**Figure 3A**) and a median PFS of 13.5months vs. 8.5 months (HR = 0.53 95% CI: 0.40–0.72, P < 0.001) (**Figure 3B**).

### 3.3 Exploratory subgroup analysis of survival benefits

**Figure 4:**
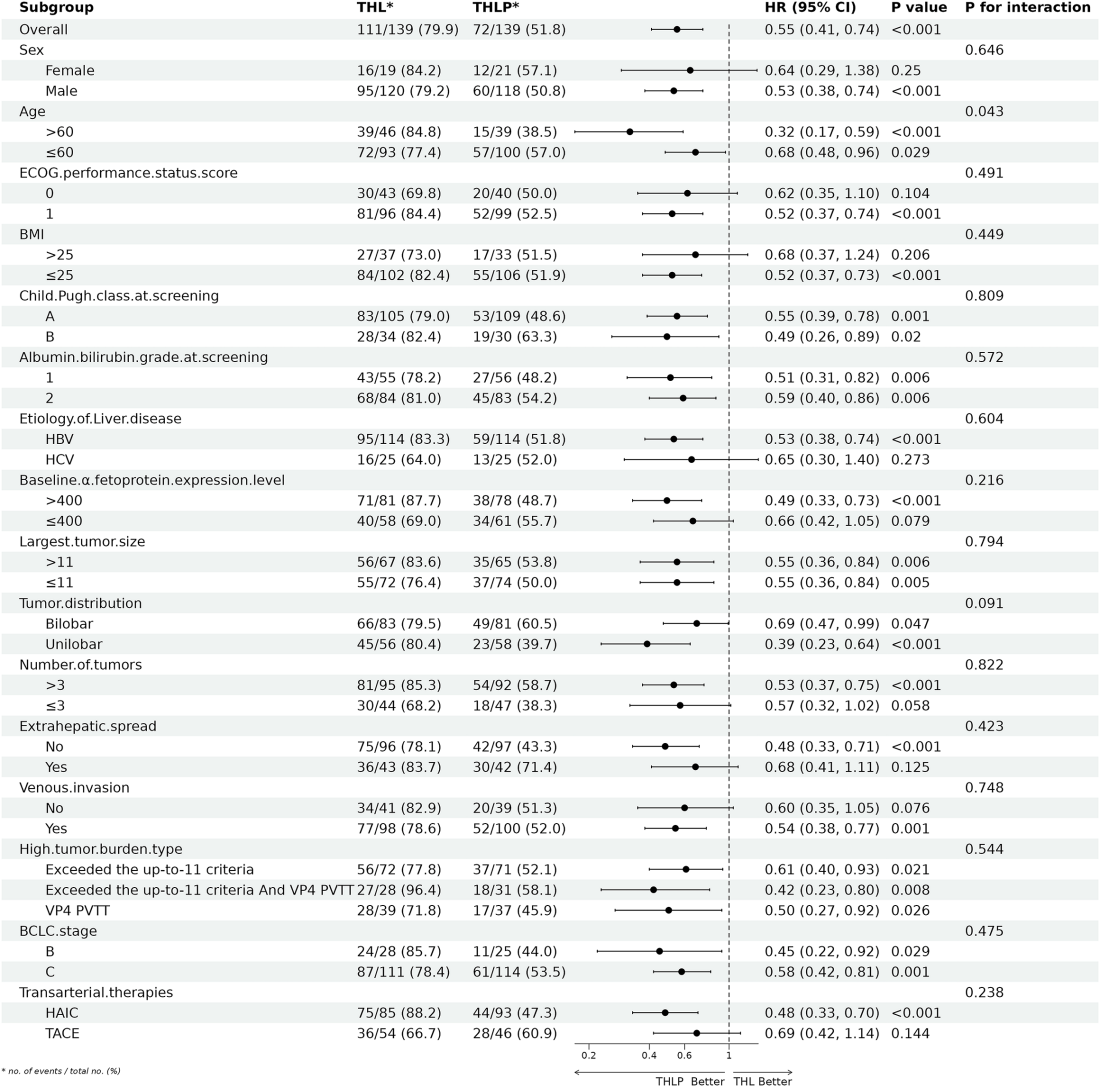
Exploratory subgroup analysis of overall survival. Forest plots confirmed the homogeneity of treatment effects across all subgroups, with no significant interaction terms (p > 0.05 for heterogeneity).

Exploratory subgroup analyses were performed to assess the consistency of the survival benefit associated with triplet therapy (THLP) across various patient demographics and disease characteristics. The results consistently favored the THLP group across all predefined subgroups. Forest plots illustrated a uniform OS (**Figure 4**) and PFS (**Supplementary Figure S2**) advantage for the THLP regimen. Of particular importance, a formal test for interaction was conducted to evaluate whether the treatment effect (THLP vs. THL) differed based on the type of locoregional therapy administered (TACE vs. HAIC). This analysis revealed no significant interaction effect (P for interaction = 0.238), indicating that the magnitude of survival benefit from the addition of a PD-1 inhibitor was not statistically different between patients who received TACE and those who received HAIC (**Figure 4**).​ This finding supports the consistency of the treatment effect across the two locoregional therapy strategies included in this study. Similarly, the survival advantage of THLP was maintained regardless of age, sex, ECOG performance status, baseline liver function (Child-Pugh class, ALBI grade), tumor burden characteristics (largest tumor size, number of tumors, distribution, presence of extrahepatic spread or venous invasion), and baseline AFP level (all P for interaction > 0.05). However, it is important to note that these analyses are exploratory and not adjusted for multiple comparisons. The small sample size in certain subgroups, such as patients with BCLC stage B disease, results in limited statistical power and wide confidence intervals; therefore, findings in these specific subsets must be interpreted with extreme caution.

### 3.4 Prognostic factors for OS in the THLP group

Multivariate cox regression identified ALBI grade 1 at screening (HR = 0.32, 95% CI: 0.17–0.62, *P* < 0.001) and unilateral tumor distribution (HR = 0.13, 95% CI: 0.06–0.27, *P* < 0.001) as independent predictors of prolonged OS (**Table 2**). Patients with ALBI grade 1 at screening achieved a median OS of 36.2 months compared to 15.2months for ALBI grade 2 at screening (**Supplementary Figures 3A**). Significant OS differences were also observed between unilateral tumor distribution vs. bilateral tumor distribution (38.6 months vs. 15.0 months) (**Supplementary Figure 3B**). No survival disparity was noted among patients with different PD-1 inhibitors (**Supplementary Figure 3C**) and trans-arterial therapies (**Supplementary Figure 3D**).

**Table 2:** Univariate and multivariate analysis of influencing factors for overall survival in the THLP group.

Characteristic	Univariable					Multivariable				
	N	Event N	HR	95% CI	*P*-value^1^	N	Event N	HR	95% CI	*P*-value^1^
Sex										
Female	21	12	—	—		21	12	—	—	
Male	118	60	0.67	0.36, 1.25	0.205	118	60	0.81	0.40, 1.64	0.561
Age (year)										
>60	39	15	—	—		39	15	—	—	
≤60	100	57	1.63	0.92, 2.89	0.092	100	57	1.13	0.58, 2.20	0.718
ECOG performance status score										
1	99	52	—	—		99	52	—	—	
0	40	20	1.12	0.66, 1.88	0.679	40	20	0.94	0.47, 1.85	0.852
BMI (kg/m²)										
>25	33	17	—	—		33	17	—	—	
≤25	106	55	1.02	0.59, 1.76	0.942	106	55	0.77	0.41, 1.44	0.412
Etiology of Liver disease										
HBV	114	59	—	—		114	59	—	—	
HCV	25	13	0.95	0.52, 1.74	0.869	25	13	0.87	0.43, 1.74	0.686
Albumin bilirubin grade at screening										
2	83	48	—	—		83	48	—	—	
1	56	24	0.23	0.14, 0.40	<0.001***	56	24	0.32	0.17, 0.62	<0.001***
Child Pugh class at screening										
B	30	19	—	—		30	19	—	—	
A	109	53	0.68	0.40, 1.16	0.155	109	53	0.96	0.53, 1.74	0.886
Baseline α fetoprotein expression level (ng/mL)										
>400	78	38	—	—		78	38	—	—	
≤400	61	34	1.02	0.64, 1.63	0.930	61	34	1.39	0.79, 2.44	0.253
Largest tumor size (cm)										
>11	65	35	—	—		65	35	—	—	
≤11	74	37	0.74	0.47, 1.18	0.209	74	37	0.70	0.37, 1.33	0.277
Tumor distribution										
Bilobar	81	53	—	—		81	53	—	—	
Unilobar	58	19	0.15	0.08, 0.28	<0.001***	58	19	0.13	0.06, 0.27	<0.001***
Number of tumors										
>3	92	54	—	—		92	54	—	—	
≤3	47	18	0.64	0.37, 1.09	0.099	47	18	1.35	0.70, 2.61	0.364
Extrahepatic spread										
Yes	42	27	—	—		42	27	—	—	
No	97	45	0.82	0.51, 1.32	0.410	97	45	0.75	0.41, 1.36	0.343
Venous invasion										
Yes	100	52	—	—		100	52	—	—	
No	39	20	1.04	0.62, 1.76	0.873	39	20	0.76	0.32, 1.83	0.544
High tumor burden type										
Exceeded the up-to-11 criteria And VP4 PVTT	31	18	—	—		31	18	—	—	
VP4 PVTT	37	17	0.48	0.24, 0.93	0.030*	37	17	0.86	0.35, 2.11	0.746
Exceeded the up-to-11 criteria	71	37	0.67	0.38, 1.18	0.164	71	37	0.86	0.41, 1.81	0.697
BCLC stage										
C	114	61	—	—		114	61	—	—	
B	25	11	0.77	0.40, 1.46	0.417	25	11	0.84	0.29, 2.44	0.744
Trans-arterial therapies										
TACE	46	28	—	—		46	28	—	—	
HAIC	93	44	0.91	0.57, 1.46	0.696	93	44	0.92	0.46, 1.82	0.806
PD-1 inhibitors type										
Camrelizumab	86	53	—	—		86	53	—	—	
Sintilimab	26	10	0.62	0.31, 1.21	0.160	26	10	0.47	0.22, 1.00	0.051
Tislelizumab	27	9	0.65	0.32, 1.32	0.230	27	9	0.52	0.21, 1.28	0.155

^1^*p<0.05; **p<0.01; ***p<0.001.

CI, Confidence Interval; HR, Hazard Ratio; BMI, Body mass index; ECOG performance status score, Eastern Cooperative Oncology Group Performance Status; HAIC, Hepatic Arterial Infusion Chemotherapy; TACE, Transarterial Chemoembolization; PVTT, Portal Vein Tumor Thrombus; HBV, Hepatitis B Virus; HCV, Hepatitis C Virus;

n = 139; N events = 72.0; statistic.log = 80.4; p.value.log = 0.000; statistic.sc = 79.7; p.value.sc = 0.000; statistic.wald = 61.4; p.value.wald = 0.000; statistic.robust = NA; p.value.robust = NA; R² = 0.439; r.squared.max = 0.988; c-index = 0.809; c-index SE = 0.022; Log-likelihood = -265; AIC = 567; BIC = 611; No. Obs. = 72.0.

### 3.5 Treatment response and correlation with survival

**Table 3** compares the short-term and long-term treatment responses before and after PSM between the two groups. The THLP group exhibited a significantly higher ORR than the THL group (73.0% vs. 53.5%, P < 0.001 before PSM and 72.7% vs. 52.5%, P < 0.001 after PSM). Notably, objective response (CR/PR) correlated with improved OS in both groups before and after PSM. Before PSM, THLP therapy responders had a median OS of 27.4 months vs. 9.0 months for non-responders (HR = 0.19, 95% CI: 0.13–0.28, P < 0.001) (**Supplementary Figure 4A**). After PSM, THLP therapy responders had a median OS of 25.7 months vs. 9.1 months for non-responders (HR =0.24, 95% CI:0.14-0.39, P < 0.001) (**Supplementary Figure 4B**). Similar trends were observed in the THL group (before PSM: HR = 0.46, 95% CI: 0.33–0.65, P < 0.001; after PSM: HR = 0.49, 95% CI:0.33-0.73, P < 0.001) (**Supplementary Figures 4C, D**).

**Table 3:** Best tumor response evaluated by mRECIST and survival outcomes before and after PSM.

mRECIST	Before PSM		*P*-value	After PSM		*P*-value
	THL, N = 187^1^	THLP, N = 248^1^		THL, N = 139^1^	THLP, N = 139^1^	
CR,n (%)	6 (3.2)	13 (5.2)		5 (3.6) 68(48.9) 60 (43.2) 6 (4.3)	7 (5.0) 94 (67.6) 27 (19.4) 11 (7.9)	
PR,n (%)	94 (50.3)	168 (67.7)				
SD,n (%)	79 (42.2)	50 (20.2)				
PD,n (%)	8 (4.3)	17 (6.9)				
ORR,n (%)	100 (53.5)	181 (73.0)	<0.001^2^	73 (52.5)	101 (72.7)	<0.001^2^
DCR,n (%)	179 (95.7)	231 (93.1)	0.253^2^	133 (95.7)	128 (92.1)	0.211^2^
PFS (mo)	8.5 (7.1–10.0)	12.8 (10.7–18.5)	<0.001^3^	8.5 (6.9–10.0)	13.5 (9.9–19.1)	<0.001^3^
OS (mo)	17.7 (16.1–21.4)	22.3 (20.3–27.4)	<0.001^3^	17.6 (15.7–21.4)	22.4 (21.6-32.4)^3^	<0.001^3^
6-mo PFS(%)	64.6	75.5		65.1	74.4	
12-mo PFS(%)	36.1	51.4		33.2	51.2	
18-mo PFS(%)	21.1	42.8	<0.001^2^	18.8	42.3	<0.001^2^
12-mo OS(%)	68.8	76.1		69.0	75.3	
18-mo OS(%)	49.8	60.5		47.9	65.0	
24-mo OS(%)	34.6	46.3	<0.001^2^	34.6	48.6	<0.001^2^

^1^n (%).

^2^Pearson's Chi-squared test.

^3^Kaplan-Meier test.

mRECIST, modified Response Evaluation Criteria in Solid Tumors; PSM, Propensity Score Matching.

CR, complete response; PR, partial response; SD, stable disease; PD, progressive disease; ORR, objective response rate; DCR, disease control rate; THL, Transarterial Chemoembolization Or Hepatic Arterial Infusion Chemotherapy combined with Lenvatinib; THLP, Transarterial Chemoembolization Or Hepatic Arterial Infusion Chemotherapy combined with Lenvatinib and programmed death 1 inhibitors.

### 3.6 Subsequent treatments

Following disease progression or intolerance to first-line therapy, second-line systemic therapies were administered to 39.6% (55/139) of patients in the THLP group and 32.4% (45/139) in the THL group. The specific types and details of these second-line regimens are summarized in **Supplementary Table S3**. Notably, a subset of patients in both groups achieved significant tumor downstaging, allowing for curative-intent surgical resection. Among these, 10 patients (15.4% of those receiving second-line therapy) in the THLP group and 1 patient (2.0%) in the THL group subsequently underwent hepatectomy. **Supplementary Table S4** and **Supplementary Figures 5**-**15** provides comprehensive details of these cases, including pretreatment imaging, post-therapy histopathology, and longitudinal survival data.

### 3.7 Change of liver function

**Figure 5:**
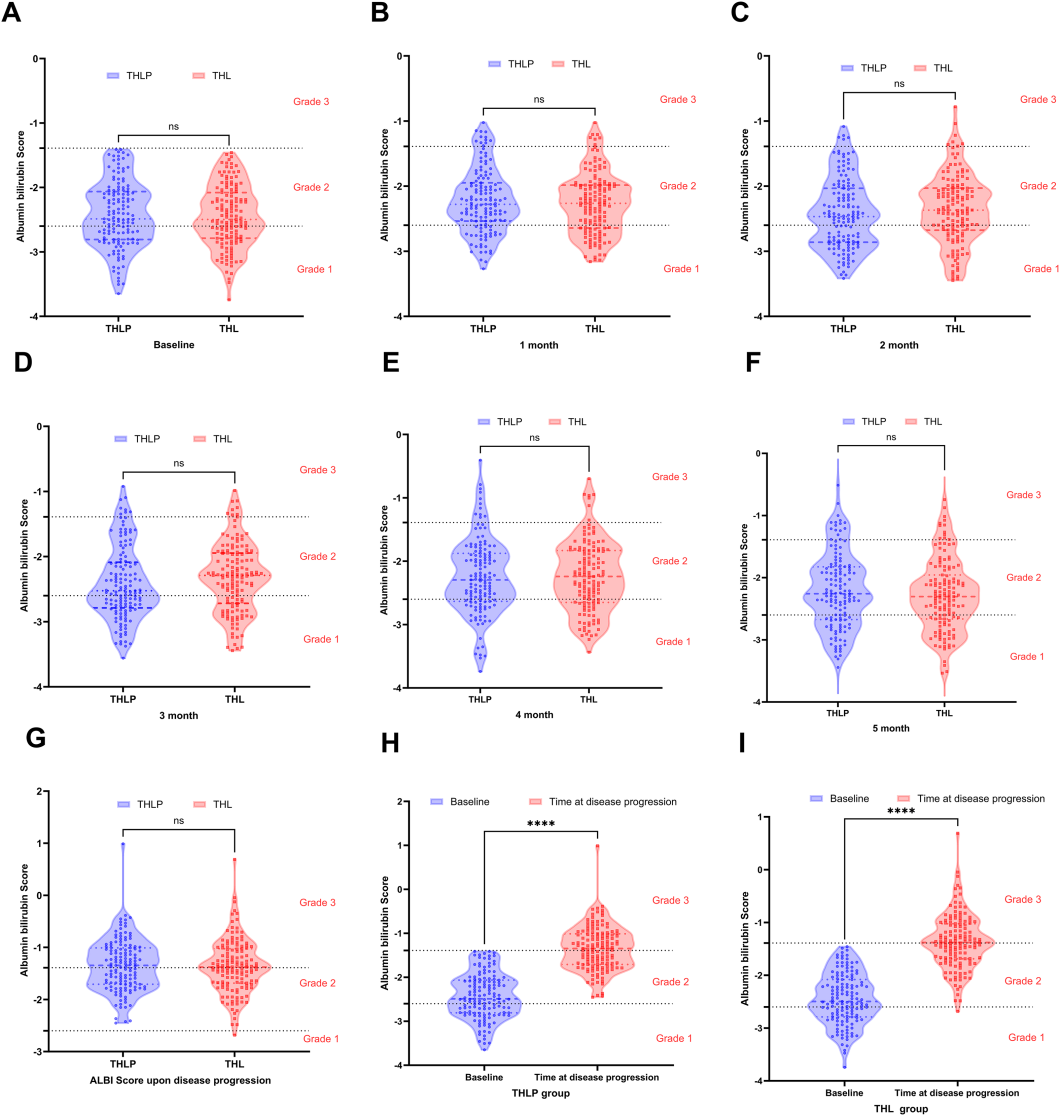
Violin plots of albumin-bilirubin (ALBI) grade dynamics across serial time points. **(A–F)** Comparison of ALBI scores between the THLP (transarterial chemoembolization/hepatic arterial infusion chemotherapy + lenvatinib + PD-1 inhibitors, blue) and THL (THLP without PD-1 inhibitors, red) groups at baseline **(A)**, 1 month **(B)**, 2 months **(C)**, 3 months **(D)**, 4 months **(E)**, and 5 months **(F)**. Grade 1–3 categories are marked with dashed horizontal lines. Statistical significance is indicated by “ns” (not significant) above group comparisons. **(G)**: ALBI scores at disease progression for both groups, showing no significant difference (ns). **(H, I)** Longitudinal comparison within the THLP **(H)** and THL **(I)** groups between baseline and disease progression timepoints, with significant ALBI deterioration in both arms (****, p < 0.001).

Serial ALBI assessments were conducted at protocol-defined landmark timepoints—specifically at baseline, monthly during the critical initial treatment phase (months 1-5), and at radiological progression—to evaluate dynamic hepatic functional changes. Comparative analysis between the THL group and THLP group revealed no statistically significant differences in ALBI scores at any timepoint (all P > 0.05, **Supplementary Table S5**; **Figures 5A–G**). However, both cohorts exhibited significant deterioration in liver function at the time of radiological progression compared to baseline (mean ΔALBI: +1.14 in THLP group, **Figures 5H**; +1.12 in THL group, **Figures 5I**; P < 0.001 for both). Subsequent univariate and multivariate logistic regression analyses identified that preservation of Child-Pugh class (absence of deterioration) at 6 months post-treatment was an independent predictor of objective response (adjusted OR: 8.71; 95% CI: 4.93–15.40; P< 0.001; **Supplementary Table S6**).

### 3.8 Cause of death

At the final follow-up, 187 patients (92 in the THL group, 95 in the THLP group) had died, with causes of death documented in 98 cases. Among these, liver failure (encompassing hepatic encephalopathy, variceal hemorrhage, hepatorenal syndrome, or refractory ascites) was the predominant cause in both cohorts, accounting for 43 of 47 deaths (91.5%) in the THL group and 49 of 51 deaths (96.1%) in the THLP group (P = 0.423) (**Supplementary Table S7**). Complications from extrahepatic metastases represented the second leading cause, responsible for 4 deaths in THL and 1 death in THLP. 1 patient in THLP group died of COVID 19.

### 3.9 Safety

The overall AE rate was -95.7% in the THLP group versus 93.5% in the THL group, with no statistically significant difference in either total AE occurrence (-P=0.597) or grade 3–4 AEs (46.8% vs. 43.2%, -P=0.629). The most frequent AEs in the THLP group were Increased ALT (46.8%), Increased AST (46.8%) and hypertension (46.8%), whereas the THL group predominantly experienced hypertension (41.7%), TACE or HAIC related AEs (31.7%), and transaminase elevation (30.2%). Notably, the THLP group exhibited significantly higher rates of Increased AST (P = 0.007), Increased ALT (P = 0.007), abdominal pain (P = 0.044), and Pyrexia (p<0.001), though the incidence of grade 3–4 events for these specific AEs did not differ between groups. Immune-related AEs (e.g., RCCEP, hypothyroidism) were exclusive to the THLP group (44.6%). No treatment-related deaths occurred (**Supplementary Table S8**). Regarding the impact of Grade 3–4 AEs on treatment delivery, permanent discontinuation of any treatment component due to toxicity occurred in-19.4% (27/139) of patients in the THLP group and 14.4% (20/139) in the THL group (P = 0.337). Dose reduction of lenvatinib was required in 28.8% (40/139) of THLP patients and 25.9% (36/139) of THL patients (P=0.687). Prolonged hospitalization (defined as an extension of stay primarily for AE management beyond the typical procedural duration) was necessitated for 17.3% (24/139)of patients in the THLP cohort and 13.7% (19/139) in the THL cohort (P=0.507). No treatment-related deaths occurred.

## 4 Discussion

This PSM study demonstrates that first-line triple therapy combining TACE or HAIC, lenvatinib, and a PD-1 inhibitor significantly improves OS, PFS, and ORR compared to TACE or HAIC plus lenvatinib in patients with HTB-uHCC. Crucially, serial ALBI assessments revealed comparable liver function trajectories during the critical initial months 1-5 (all P>0.05), providing the first systematic evidence that intensifying therapy with PD-1 inhibitors does not exacerbate early hepatic injury in this vulnerable population.

A central challenge in managing this aggressive disease is the rapid hepatic functional deterioration following progression (14, 16, 17), which severely restricts eligibility for subsequent therapies (18) – a challenge underscored here, as over half of patients progressing in both cohorts developed ALBI grade 3, precluding further systemic treatment. Consistent with previous study (16, 19), our findings further elucidate that responders consistently maintained superior liver function at landmark assessments compared to non-responders. Importantly, the significantly longer OS observed in responders versus non-responders highlights the profound clinical impact of achieving tumor control, as it directly translates to survival benefit. Multivariate analysis reinforced this link, identifying the preservation of Child-Pugh class (absence of deterioration) at 6 months as a strong independent predictor of ORR (16, 19). This compellingly associates effective tumor control – achieved notably more frequently with THLP (ORR 72.7%) – with the preservation of hepatic synthetic and detoxification capacity, likely by mitigating tumor-driven parenchymal destruction, vascular compression, and paraneoplastic inflammation (19). Therefore, the higher ORR afforded by THLP not only directly impacts survival but also indirectly preserves liver function, a critical determinant for both OS and the feasibility of sequential therapies (14, 16, 18, 19).

Within the THLP cohort, baseline ALBI grade 1 and unilateral tumor distribution emerged as significant independent predictors of prolonged OS, aligning with evidence that preserved hepatic reserve directly correlates with treatment tolerance and survival (20). We posit that bilateral involvement heightens liver failure risk through substantial depletion of functional liver volume, compromised portal flow (exacerbating portal hypertension), and increased treatment-related injury from diffuse disease management (21–23). Consequently, baseline ALBI grade and tumor laterality should be integrated into clinical decision-making to identify patients most likely to tolerate and benefit from aggressive first-line strategies like THLP.

The notable absence of independent prognostic significance for both extrahepatic metastasis (EHM) and alpha-fetoprotein (AFP) levels in this specific cohort with HTB-uHCC presents findings that contrast with some previous studies. This divergence can be attributed to the unique clinical characteristics and treatment context of this population. Mechanistically, the lack of prognostic value for EHM is explained by the overwhelming impact of intrahepatic disease progression. Hepatic decompensation events (such as variceal bleeding) accounted for the vast majority (93.9%) of deaths, vastly overshadowing mortality directly attributable to EHM complications (6.1%). This underscores that in this specific HTB-uHCC population, the profound intrahepatic tumor burden drives rapid functional decline and liver failure, often culminating in mortality before EHM-related morbidity becomes life-limiting. This situation is compounded by limited access to salvage therapies after disease progression, with only 39.9% of patients receiving second-line treatment. Consequently, first-line therapeutic strategies that prioritize rapid intrahepatic disease control and preservation of hepatic reserve—key strengths of the THLP (HAIC, lenvatinib, tislelizumab with/without TAE) regimen used in this study—are of paramount importance (24). Similarly, the absence of independent prognostic value for AFP levels in this cohort contrasts with reports from major trials like IMbrave150 (25) and REFLECT (12), where a baseline AFP level >400 ng/mL typically independently predicted survival. Two factors specific to this study's design and patient population likely explain this discrepancy: Skewed AFP Distribution: Patients with HTB-uHCC exhibit universally elevated AFP levels. The median AFP in this cohort was dramatically high (1,636 ng/mL), compared to a median of 89 ng/mL in the REFLECT trial population. This creates a potential ceiling effect that diminishes its discriminative power for risk stratification. Notably, 57.6% of this cohort had AFP levels exceeding 400 ng/mL, compared to approximately 35% in the IMbrave150 study. Temporal Variability: AFP was measured within 7 days prior to treatment initiation. However, the rapid tumor progression inherent to HTB-uHCC could cause significant fluctuations in AFP levels between the time of blood sampling and the actual commencement of therapy, adding noise to its prognostic value. It is important to emphasize that this finding does not negate the established prognostic utility of AFP in broader HCC populations. Rather, it highlights its context-specific limitations in cohorts with exceptionally high tumor burden receiving intensive multimodal therapy. Future clinical studies focusing on HTB-uHCC should consider incorporating longitudinal AFP measurements and potentially exploring etiology-adjusted thresholds to better define its role in this specific setting.

Furthermore, our subgroup analysis within the THLP cohort revealed that baseline ALBI grade, but not Child-Pugh class, was independently predictive of OS. This differential prognostic performance, while initially surprising, may be explained by several key considerations specific to our intensively treated, high tumor burden population. First, the Child-Pugh score incorporates subjective clinical components such as the severity of ascites and hepatic encephalopathy. In our cohort of patients with high tumor burden, the interpretation of these features can be challenging. For instance, mild ascites can be paraneoplastic or related to portal hypertension from extensive tumor involvement/Vp4 PVTT, not purely reflective of hepatic decompensation. This might introduce "noise" into the Child-Pugh assessment, diluting its correlation with survival outcomes primarily driven by cancer-specific factors in our study. Second, the patient selection for our study (Child-Pugh A and B7) created a relatively homogenous population in terms of Child-Pugh classification (likely mostly Class A and a few B7), restricting its statistical variability and thus its power to predict outcomes. The ALBI grade, with its finer gradation (e.g., ALBI grade 1 vs. 2), possessed greater discriminative power within this narrow, functionally preserved range to identify those with truly optimal liver function best suited to tolerate and benefit from intensive triplet therapy.

The significant median OS gain of 4.8 months (22.4 vs. 17.6months) and PFS gain of 5.0 months (13.5 vs. 8.5months) with triplet therapy (THLP) must be balanced against its AE profile in clinical decision-making. While the absolute incidence of severe AEs was numerically higher in the THLP group, the difference was not statistically significant, and the types of AEs were generally manageable with standard supportive care and dose modifications. This suggests that the survival benefit offered by the intensified regimen may be achieved without a proportionate increase in severe, treatment-limiting toxicity. When counseling patients with HTB-uHCC, clinicians should present this efficacy-toxicity tradeoff, highlighting the potential for meaningful survival extension while acknowledging the increased likelihood of manageable immune-related and hepatic AEs requiring closer monitoring and potential intervention.

Notwithstanding our robust propensity score-matched analysis, several limitations warrant cautious interpretation of our findings. First, this study has limitations inherent to its retrospective design and the need to create analytically viable cohorts. The pooling of two distinct locoregional therapies, TACE and HAIC, as well as three different PD-1 inhibitors, for the primary analysis could potentially mask nuanced differences in efficacy or safety between these specific modalities. Although our interaction analysis showed no statistically significant differential treatment effect between the TACE and HAIC subgroups (P for interaction = 0.238), thus providing some justification for the pooled analysis, this finding should be interpreted with caution as the study was not powered to definitively establish equivalence between these strategies. This approach was primarily driven by clinical practice, where the choice between TACE and HAIC is often tailored to individual tumor anatomy and physician judgment, and by the desire to evaluate the overarching concept of adding a PD-1 inhibitor to a backbone of locoregional therapy and lenvatinib. Similarly, the aggregation of different PD-1 inhibitors, while reflecting real-world prescribing patterns, precludes conclusions about the potential superiority of any single agent. Therefore, our results support the general strategy of first-line treatment intensification with triplet therapy for HTB-uHCC, but future research, ideally in randomized trials, is needed to determine the optimal combination of specific locoregional and immunotherapeutic agents for particular patient subsets.. Second, the etiological spectrum of our cohort was exclusively restricted to hepatitis B and C virus-related HCC. While this accurately reflects the predominant etiology in our geographic region (East Asia) and the patient population of our tertiary care center, it may limit the generalizability of our findings to populations with a different etiological landscape. Specifically, our results may not be fully applicable to patient cohorts with a high prevalence of alcohol-associated liver disease or non-alcoholic steatohepatitis (NASH), which are leading causes of HCC in Western countries. It is well-documented that the underlying etiology influences the tumor immune microenvironment (TIME) and liver immune biology. For instance, HBV-related HCC is often characterized by a more immunosuppressive TIME (26), while NASH-related HCC may involve unique immunometabolic pathways that could modulate the response to immune checkpoint inhibitors (27). Therefore, the significant benefits we observed with the addition of PD-1 inhibitors in this predominantly HBV-infected cohort might not be directly extrapolated to patients with HCC of other etiologies, who may exhibit differential efficacy and safety profiles. Future studies are warranted to validate the efficacy of this triple-therapy approach in etiologically diverse, multi-regional cohorts. Third, the heterogeneity in chemotherapeutic regimens used during TACE (e.g., anthracyclines, platinum agents, or combination therapies) represents a notable methodological limitation, as differential drug selection could influence local tumor control and systemic immune modulation. Future prospective, multicenter randomized trials with standardized treatment protocols are imperative to validate these findings and establish optimized regimens for specific HTB-uHCC subgroups. Fourth, our study has important limitations related to statistical power and methodology. As a retrospective study, the sample size for certain patient subgroups was inherently limited. Specifically, the small number of patients with BCLC stage B disease precludes any definitive conclusions regarding treatment efficacy within this subset. Furthermore, the numerous exploratory subgroup analyses conducted increase the potential for false-positive findings in the absence of adjustment for multiple comparisons. Therefore, the subgroup results presented here should be strictly viewed as hypothesis-generating, highlighting the need for validation in future prospective studies with larger, well-powered cohorts. Last critical limitation of this analysis is the lack of patient-reported outcomes (PROs) or formal quality-of-life (QoL) assessments, which precludes a comprehensive understanding of the subjective patient experience and the full impact of AEs on daily living. Future prospective studies should incorporate PROs to better quantify the net clinical benefit of treatment intensification.

In conclusion, this study establishes THLP triple therapy as a paradigm-shifting first-line approach for HTB-uHCC. By achieving superior tumor control while concurrently preserving hepatic function during the critical initial phase, THLP significantly improves survival and mitigates therapeutic attrition. Proactive toxicity management remains essential, but these findings strongly support early treatment intensification in appropriately selected patients and pave the way for biomarker-driven personalized therapy.

## Data Availability

The raw data supporting the conclusions of this article will be made available by the authors, without undue reservation.
