# Supplementary material for: Retrospective analysis of transarterial chemoembolization or hepatic arterial infusion chemotherapy combined with lenvatinib with or without PD-1 inhibitor as first-line therapy for unresectable hepatocellular carcinoma with high tumor burden: a propensity score-matched study
Source: Front Immunol. 2026 Feb 16;17:1717797. doi: 10.3389/fimmu.2026.1717797 (PMC12950717; doi:10.3389/fimmu.2026.1717797)
Supplement: Supplementary file 1 [file DataSheet1.docx]

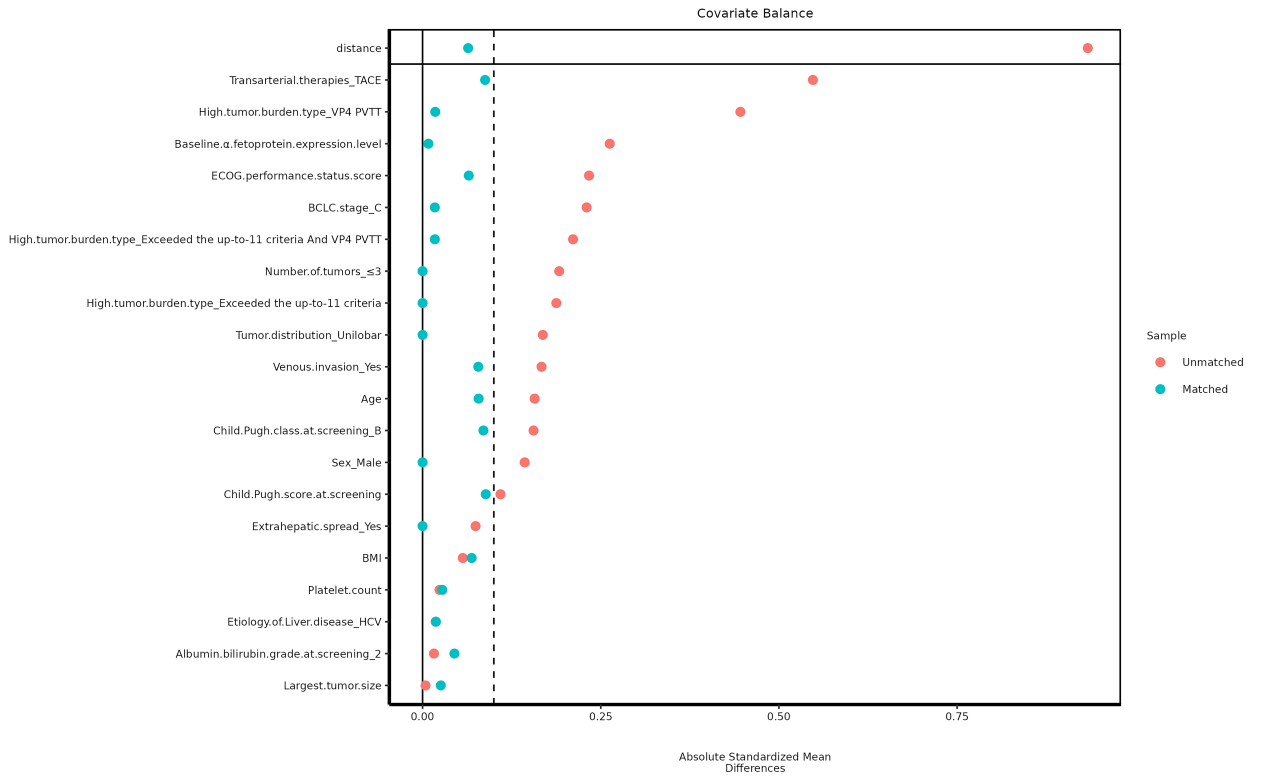


**Supplementary Figure S1.** Absolute Standardized Mean Differences for Baseline Characteristics Before (Unmatched) and After (Matched) Propensity Score Matching


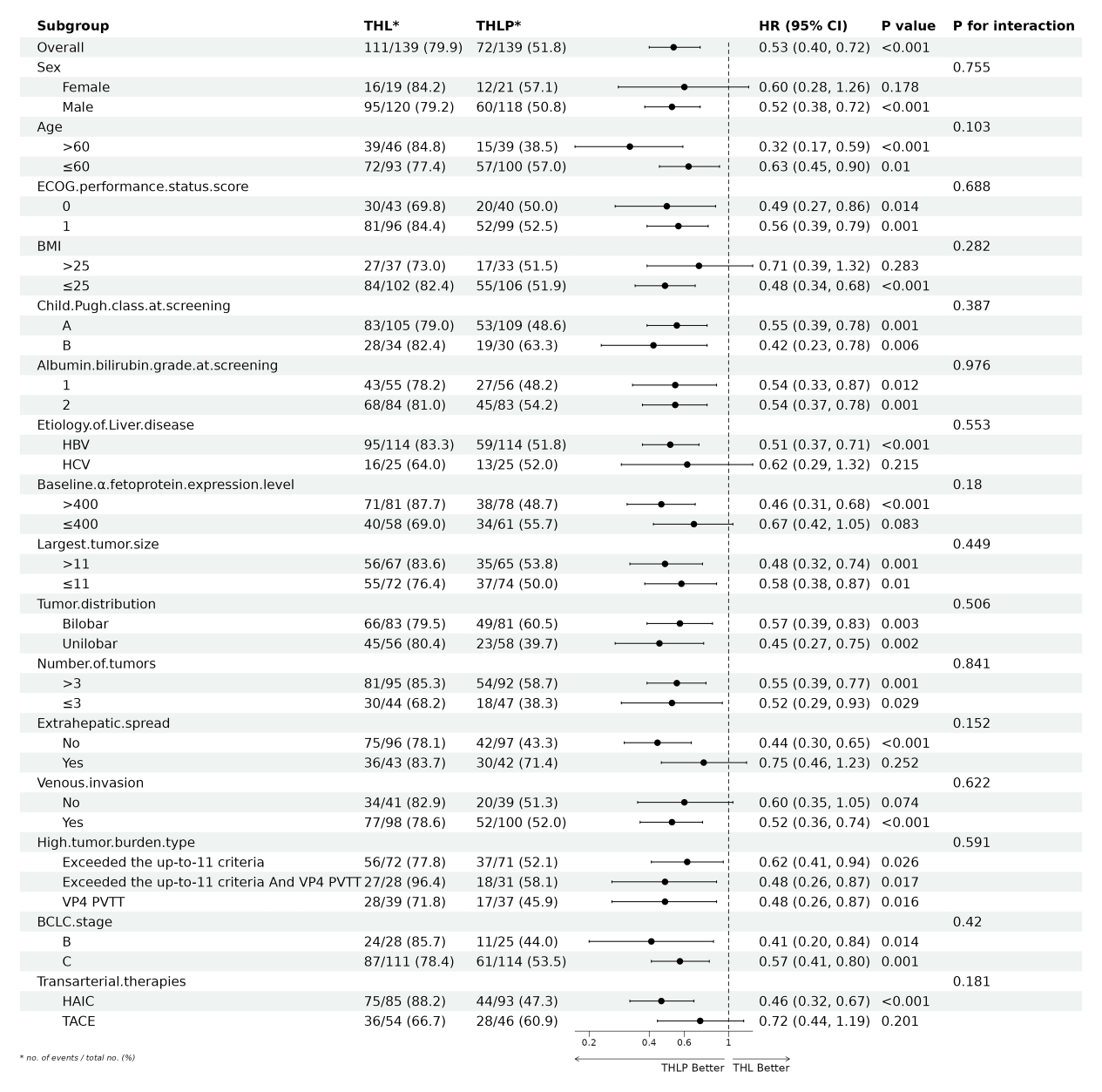


**Supplementary Figure S2.** Exploratory Subgroup Analysis of Progression-Free Survival. Forest plots confirmed the homogeneity of treatment effects across all subgroups, with no significant interaction terms (*p* > 0.05 for heterogeneity).

**Supplementary Figures S3** **A**


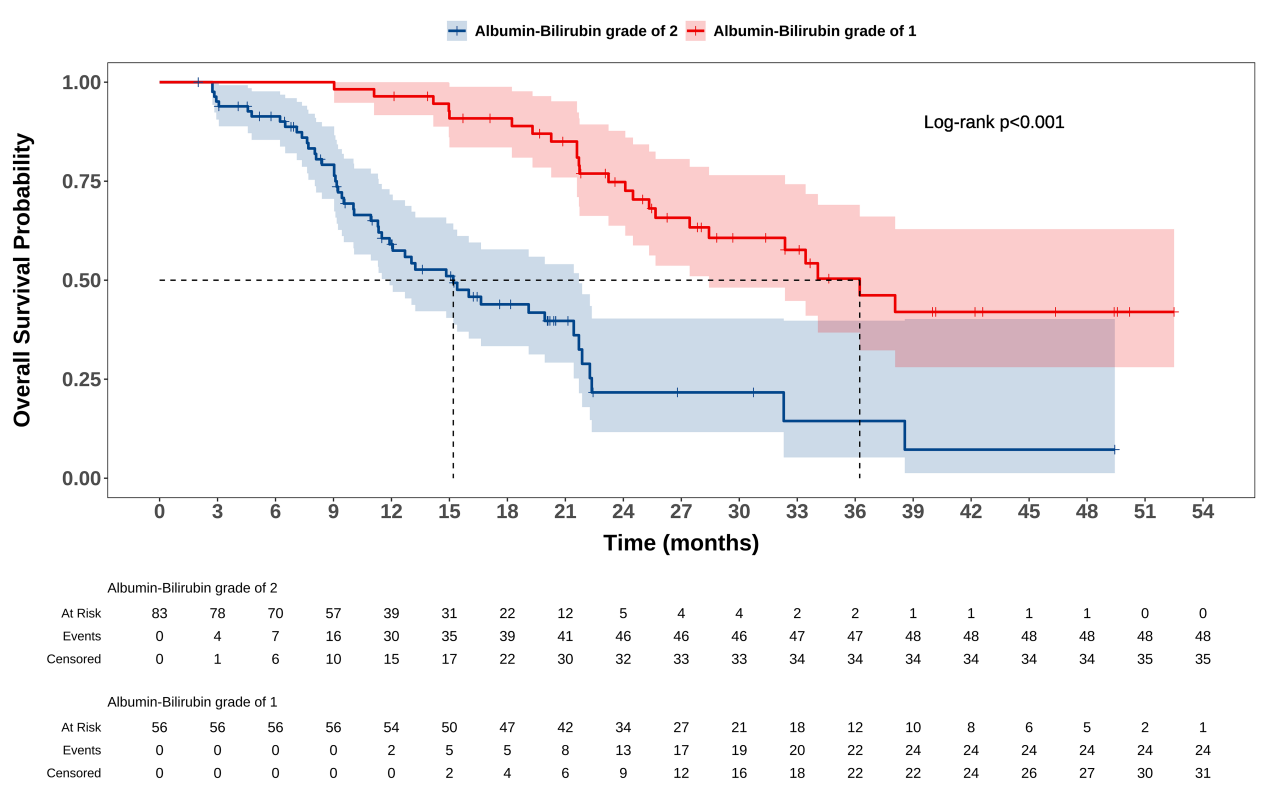


**Supplementary Figures S3** **B**


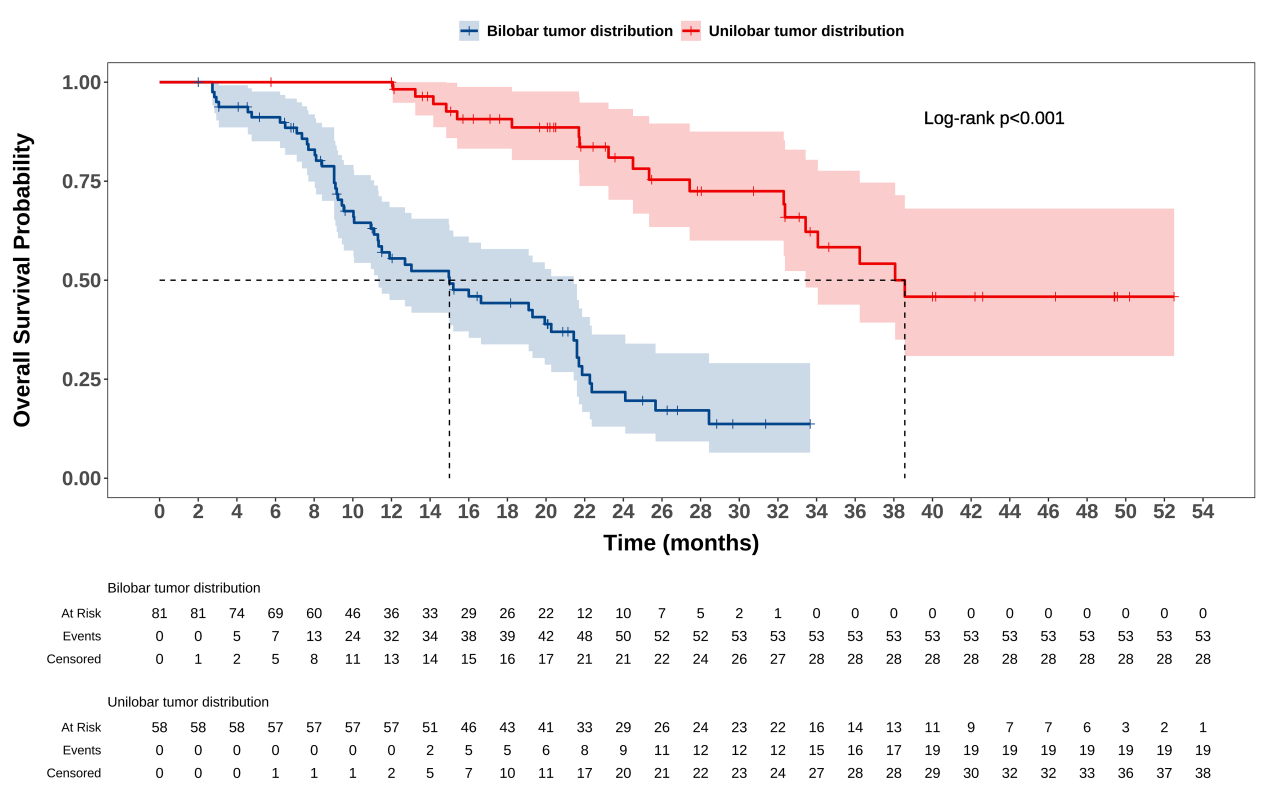


**Supplementary Figures S3** **C**


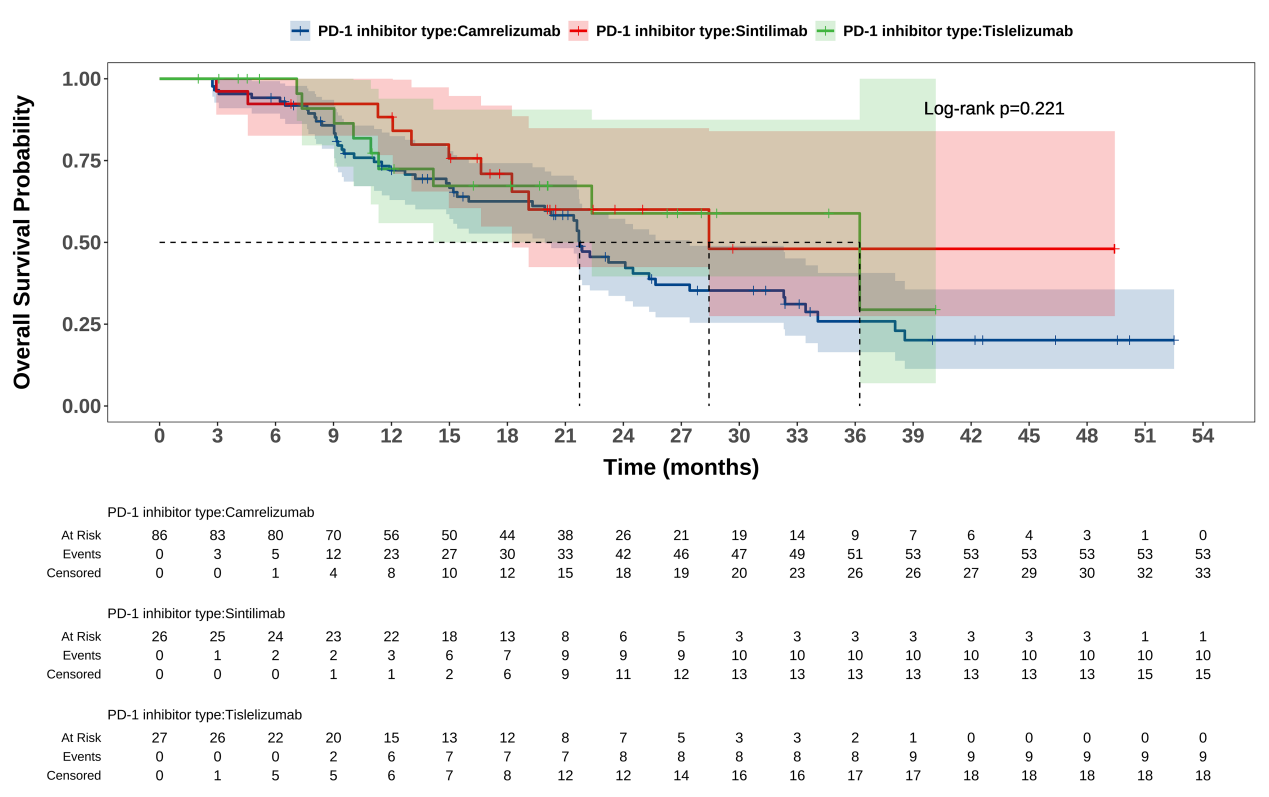


**Supplementary Figures S3** **D**


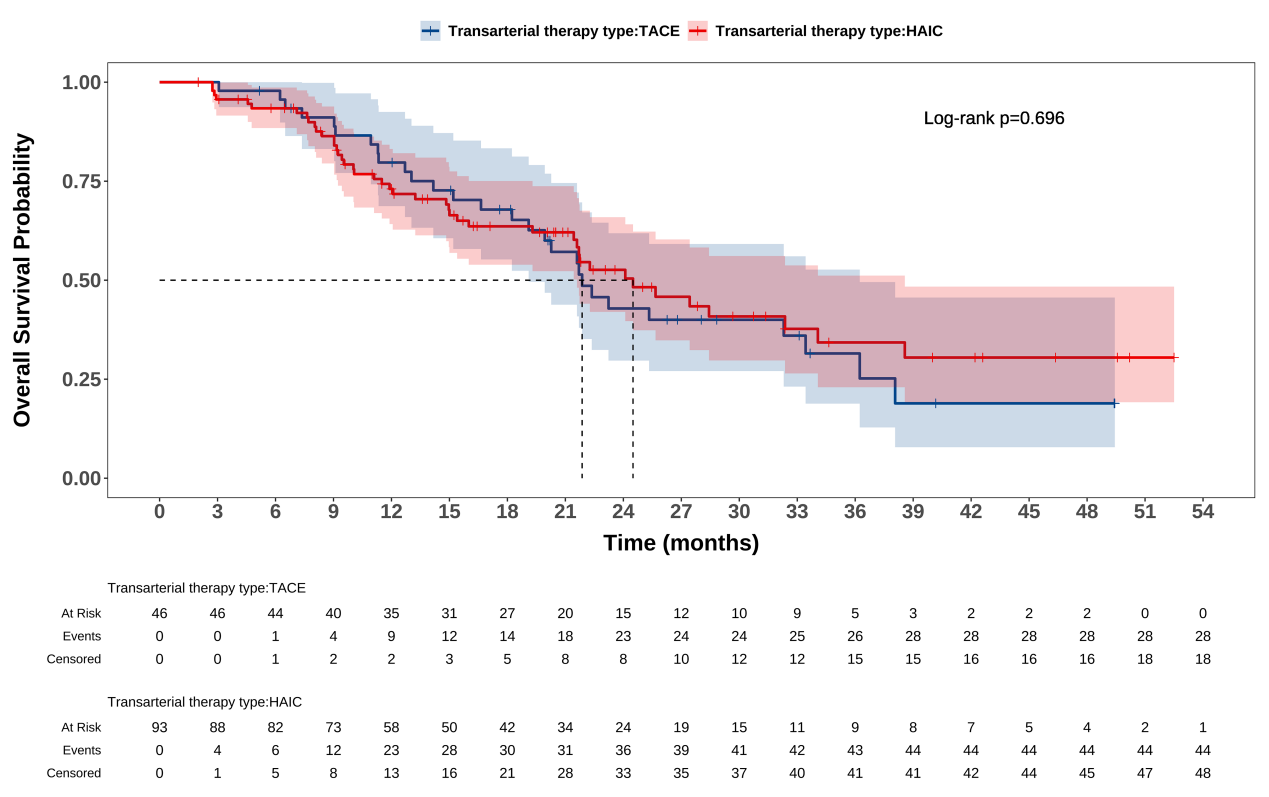


**Supplementary Figures S3.** Kaplan-Meier curves show percent overall survival of THLP group stratified by (**A**) ALBI grade, (**B**) tumor distribution, (**C**) PD-1 inhibitors and (**D**) trans-arterial therapies. **Abbreviations:** THL, Transarterial Chemoembolization Or Hepatic Arterial Infusion Chemotherapy combined with Lenvatinib and programmed death 1 inhibitors; ALBI, Albumin-bilirubin.

**Supplementary Figures S4** **A**

**
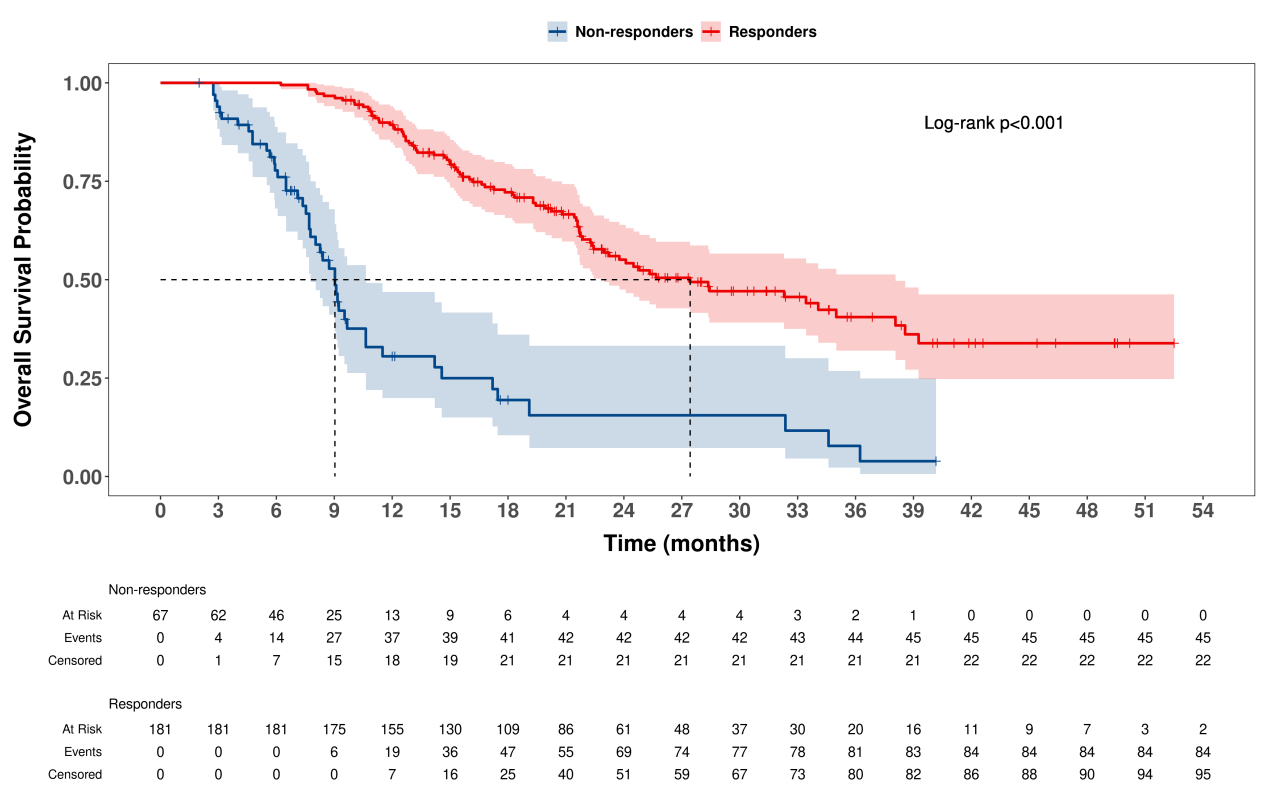
**

**Supplementary Figures S4** **B**


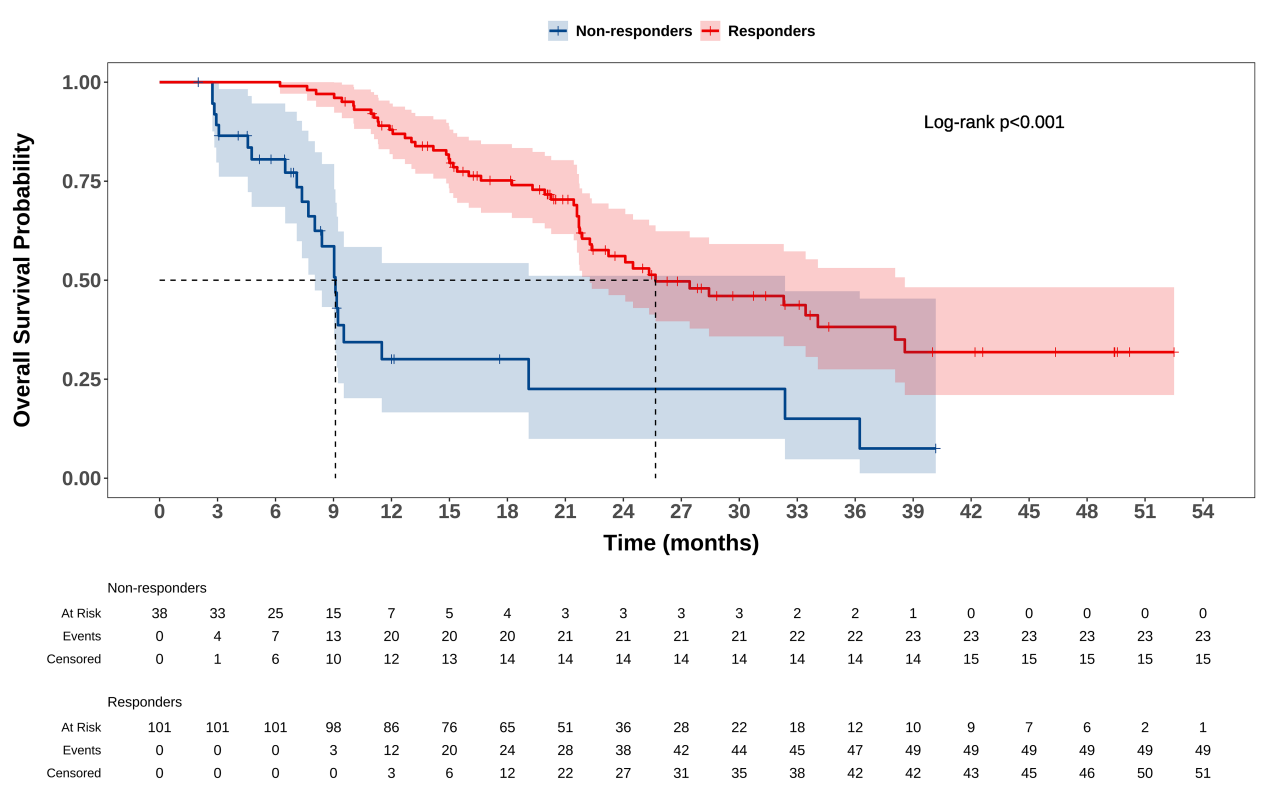


**Supplementary Figures S4** **C**


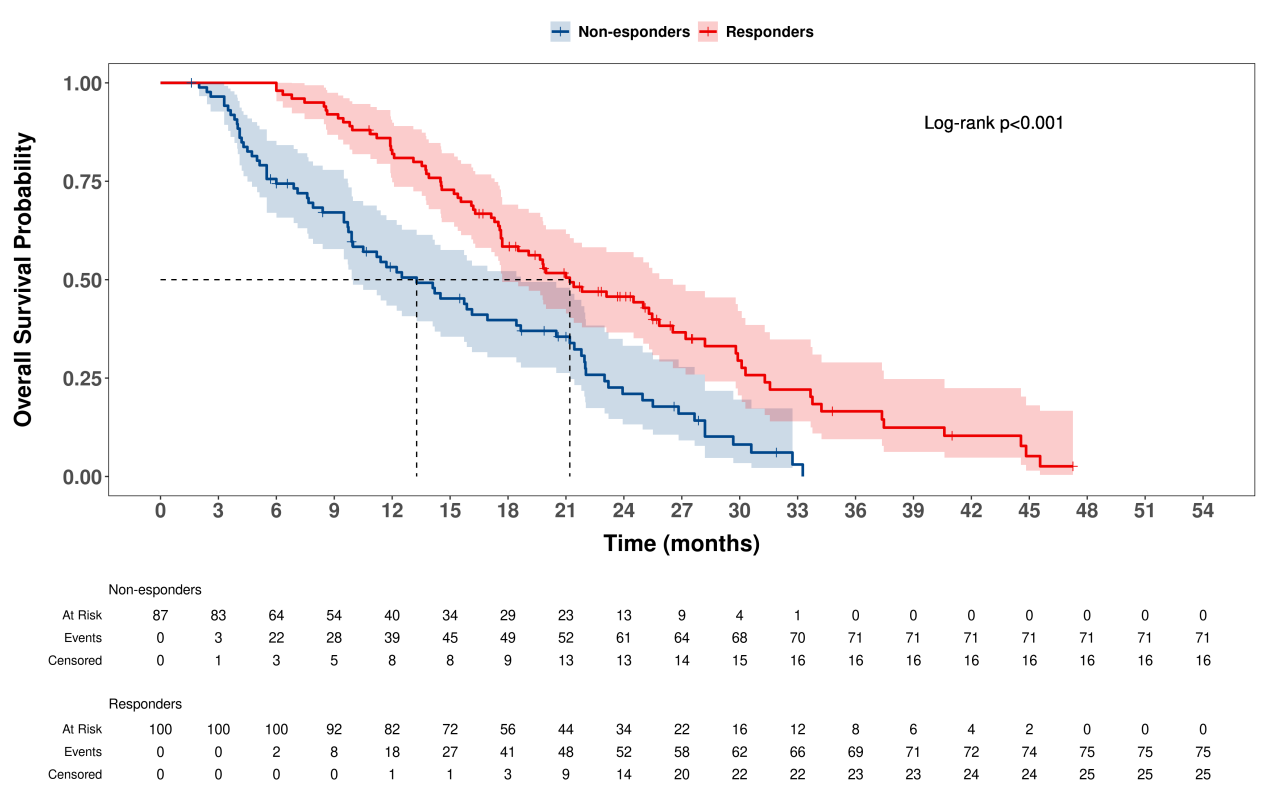


**Supplementary Figures S4 D**


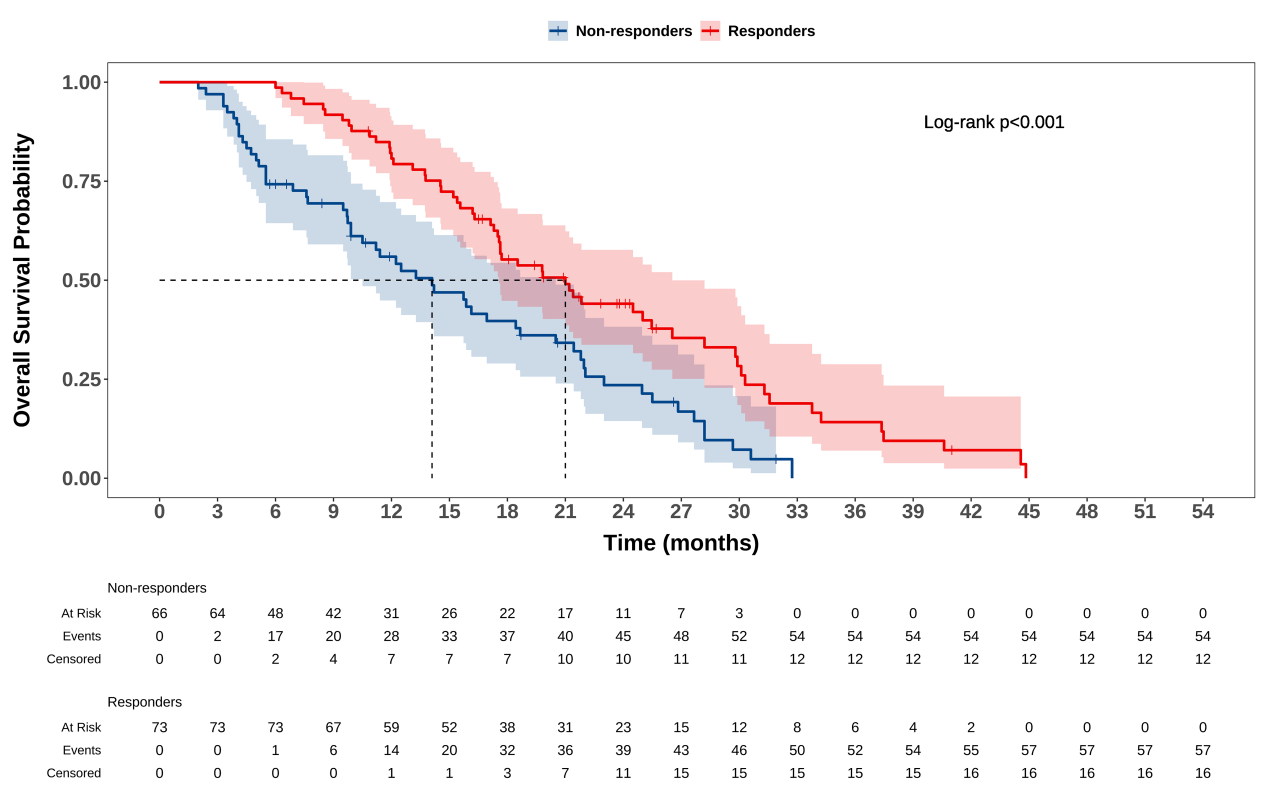


**Supplementary Figures S4.** Kaplan-Meier Curves for Overall Survival Stratified by Objective Response Status (Responders vs. Non-Responders) in the THLP and THL Groups: Pre- (**A** and **B**) and Post-Propensity Score Matching Analysis (**C**and **D**)

**Abbreviations:** THL, Transarterial Chemoembolization Or Hepatic Arterial Infusion Chemotherapy combined with Lenvatinib; THLP, Transarterial Chemoembolization Or Hepatic Arterial Infusion Chemotherapy combined with Lenvatinib and programmed death 1 inhibitors.


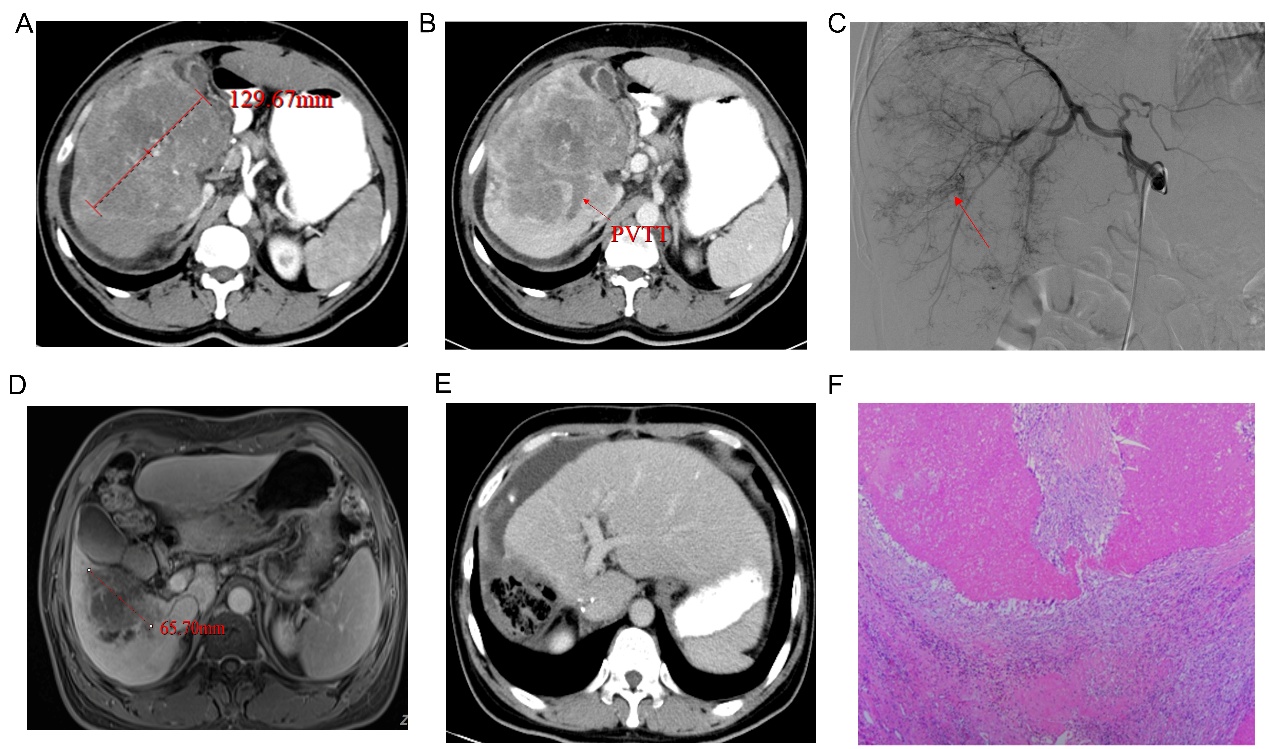


**Supplementary Figures S5.**

**Case 1.** (**A**) Pre-treatment imaging: Axial contrast-enhanced CT (arterial phase) demonstrates a large HCC lesion in the right-lobe (pre-treatment tumor maximum diameter: 12.9 cm) and Vp3 Portal Vein Tumor Thrombosis (PVTT) involving the right ranch (**B**, red arrow). (**C**)Selective hepatic arteriography demonstrates tumor staining (red arrow). (**D**) Post-conversion therapy imaging: Follow-up contrast-enhanced MRI demonstrates significant tumor shrinkage (post-treatment tumor maximum diameter: 6.6cm) (**E**)Contrast-enhanced CT at 3 months post-surgery shows no evidence of residual or recurrent disease. (**F** Histopathological findings: Extensive sampling of the submitted liver tumor and adjacent satellite lesions revealed predominantly necrotic tissue with surrounding chronic inflammatory cell infiltration and histiocytic reaction. No residual tumor tissue was identified, consistent with post-therapeutic changes. No invasion into the hepatic capsule was observed.


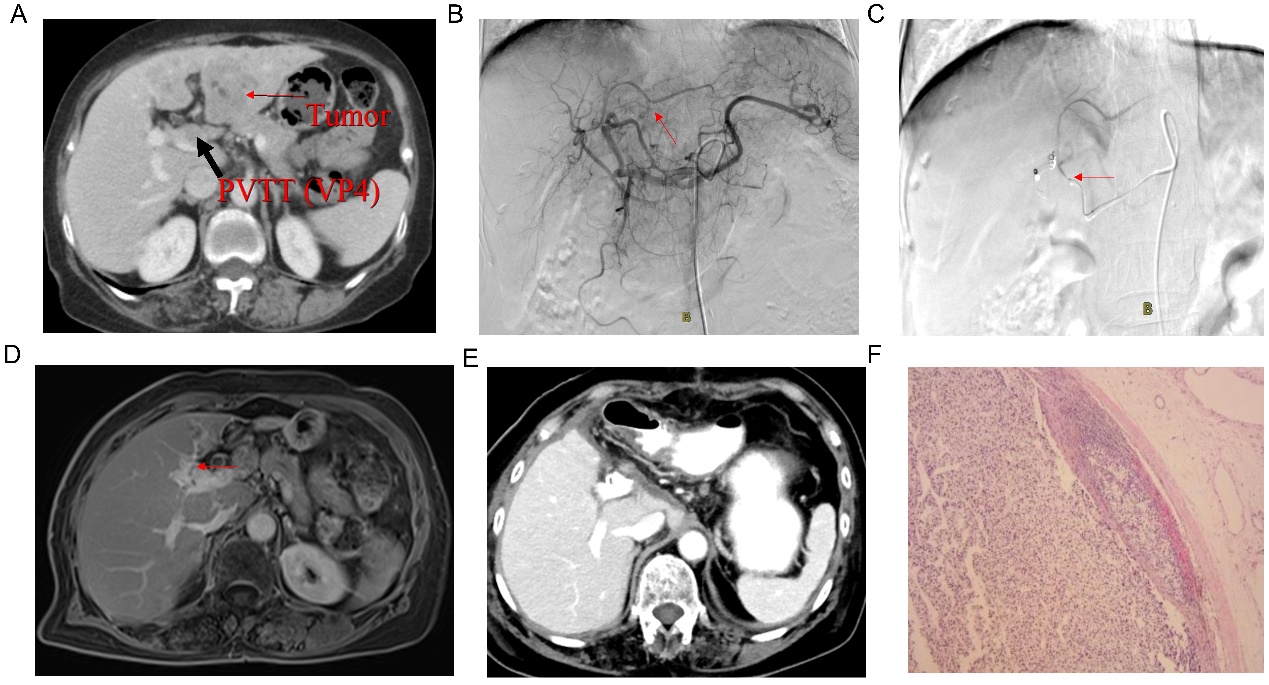


**Supplementary Figures S6.**

**Case 2.** (**A**)Pre-treatment imaging: Axial contrast-enhanced CT (venous phase) demonstrates a tumor in the left hepatic lobe (red arrow) and Vp4 portal vein tumor thrombus (PVTT) extending into the main portal vein (black bold arrow). (**B**)Selective hepatic arteriography demonstrates intense tumor staining (red arrow) and tortuous tumor-feeding arteries. (**C**)Selective left hepatic arteriography confirms microcatheter tip positioning (red arrow) for HAIC. (**D**) Post-conversion therapy imaging: Follow-up MRI (portal venous phase) shows regression of PVTT to segmental branches (red arrow). (**E**)Contrast-enhanced CT at 6months post-surgery shows no evidence of residual or recurrent disease. **(F**)Histopathological features: Extensive hemorrhagic necrosis and treatment-related alterations.


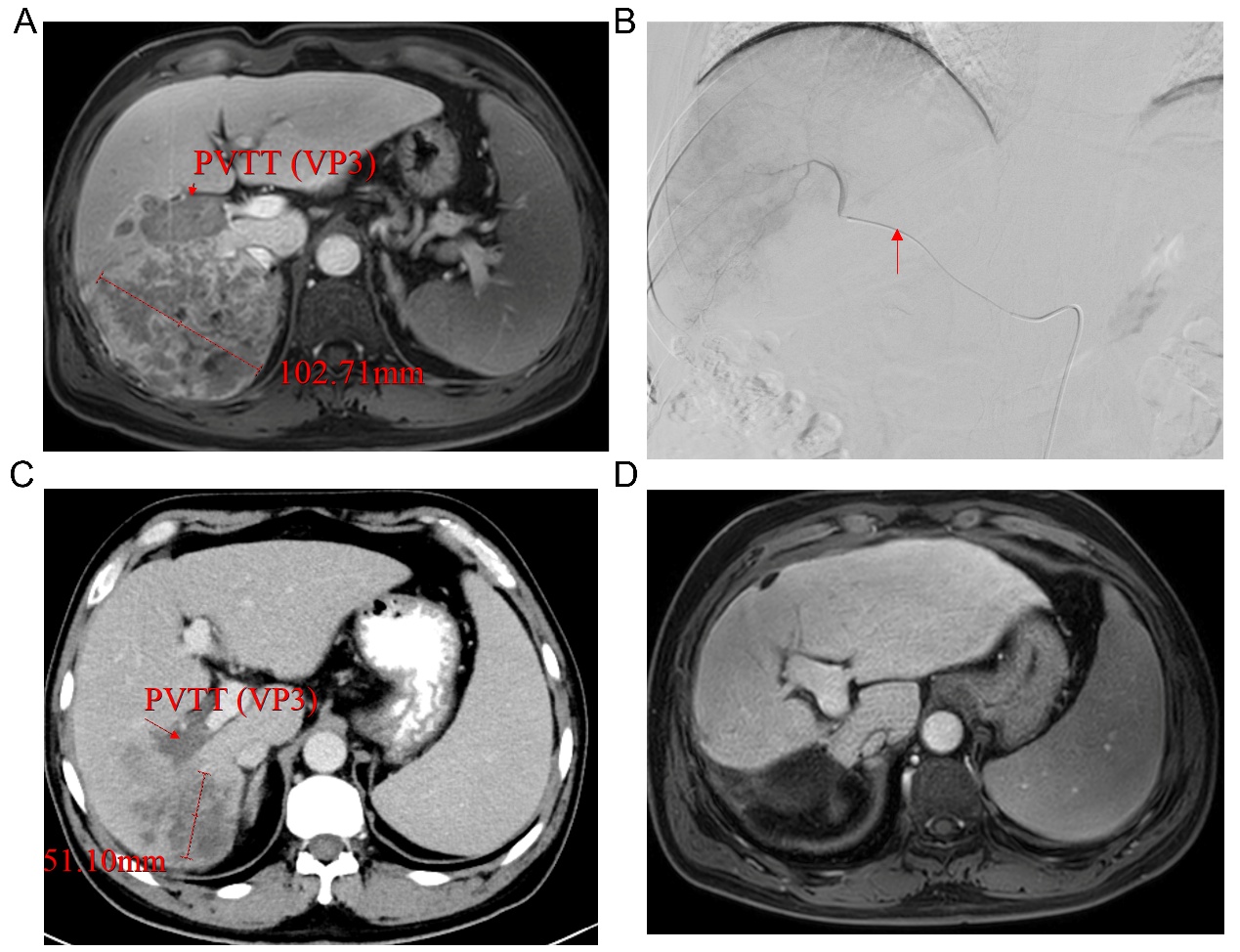


**Supplementary Figures S7.**

**Case 3. (A)** Pre-treatment imaging: Axial Contrast-enhanced MRI demonstrates a massive tumor in the right hepatic lobe (pre-treatment tumor maximum diameter: 10.3 cm) and Vp3 portal vein tumor thrombus (PVTT) in the right branch (red arrow). **(B)** Selective right hepatic arteriography confirms tumor staining and microcatheter tip positioning (red arrow) for HAIC**. (C)** Post-conversion therapy imaging: Follow-up Contrast-enhanced CT demonstrates significant tumor shrinkage (post-treatment tumor maximum diameter: 5.1 cm) in the right hepatic lobe and regression of PVTT (red arrow). **(D)** Contrast-enhanced CT at 2months post-surgery shows no evidence of residual or recurrent disease.


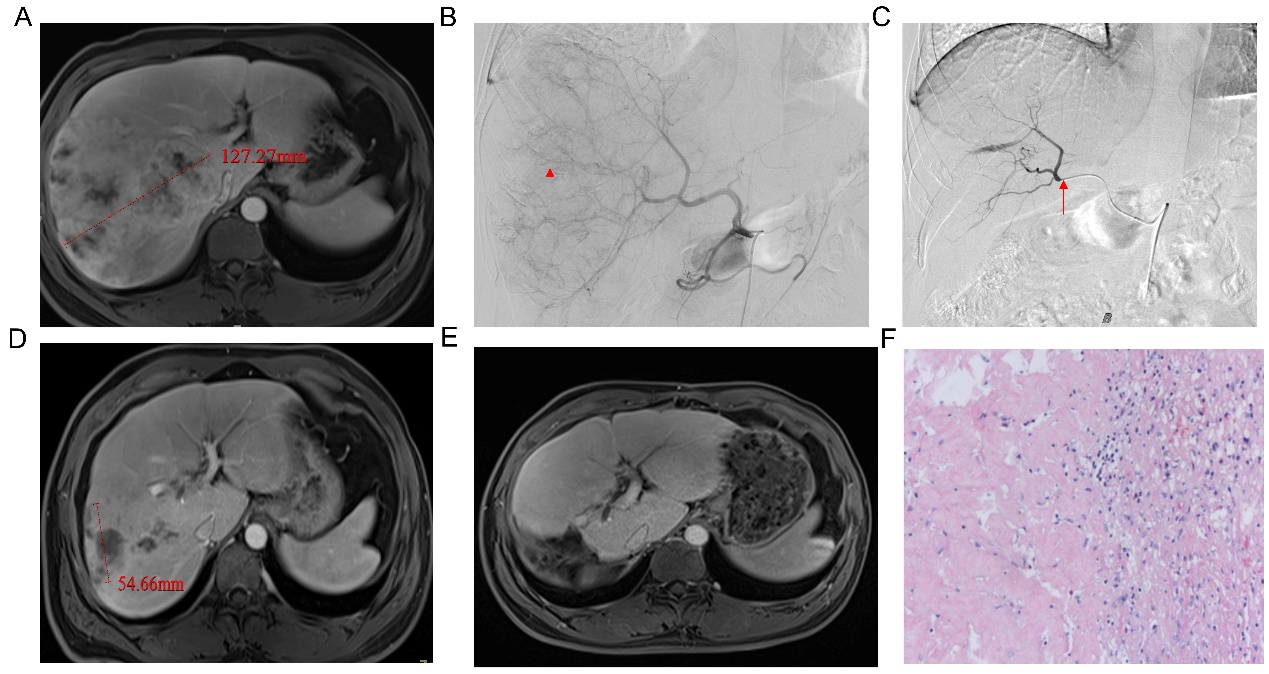


**Supplementary Figures S8.**

**Case 4.** (**A**)Pre-treatment imaging: Axial contrast-enhanced MRI (venous phase) demonstrates a large HCC lesion in the right-lobe (pre-treatment tumor maximum diameter: 12.7cm). (**B**) Selective hepatic arteriography demonstrates tumor staining (red arrow). (**C**)Selective right hepatic arteriography confirms tumor staining and microcatheter tip positioning (red arrow) for HAIC. (**D**) Post-conversion therapy imaging: Follow-up Contrast-enhanced MRI demonstrates significant tumor shrinkage (post-treatment tumor maximum diameter: 5.5 cm). (**E**) Contrast-enhanced CT at 2months post-surgery shows no evidence of residual or recurrent disease. (**F)** Histopathological findings: Findings include ​necrosis, ​fibrosis, and ​chronic inflammatory cell infiltration, consistent with ​post-therapeutic changes.


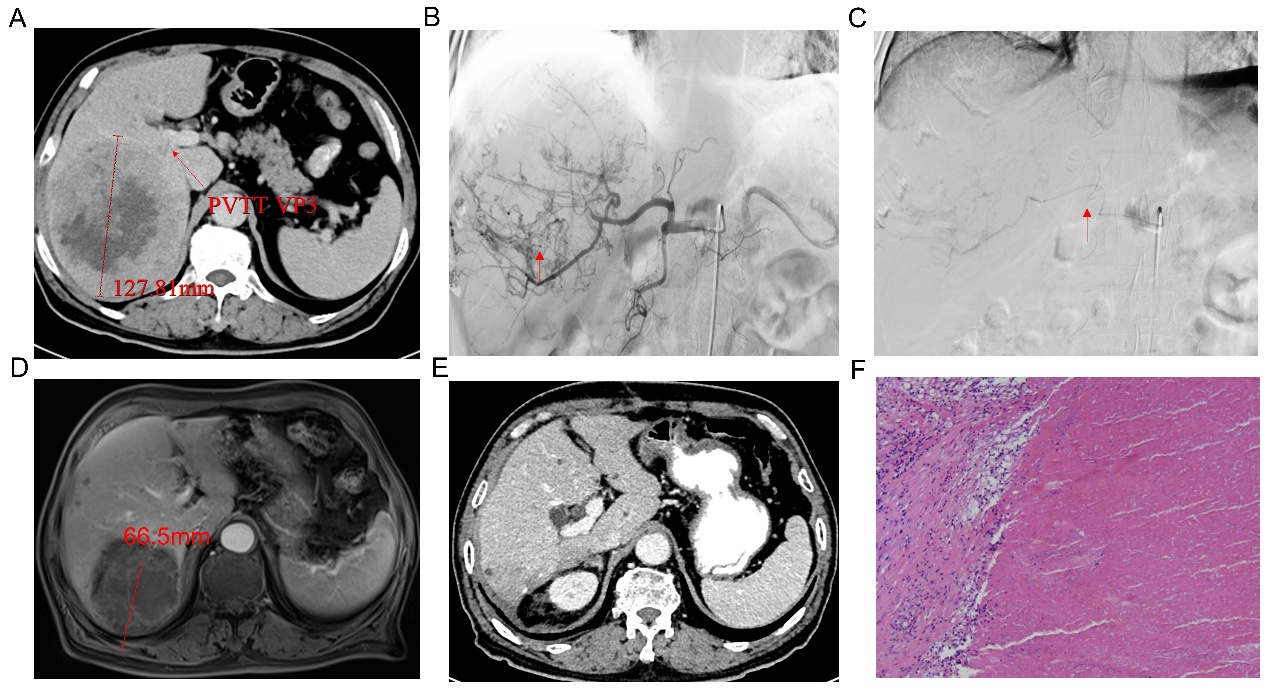


**Supplementary Figures S9.**

**Case 5.** (**A**) Pre-treatment imaging: Axial contrast-enhanced CT (venous phase) demonstrates a large HCC lesion in the right-lobe (pre-treatment tumor maximum diameter: 12.8cm) and Vp3 PVTT (red arrow). (**B**)Selective hepatic arteriography demonstrates tumor staining (red arrow). (**C**) Selective right hepatic arteriography confirms tumor staining and microcatheter tip positioning (red arrow) for HAIC

(**D**) Post-conversion therapy imaging: Follow-up Contrast-enhanced MRI demonstrates significant tumor shrinkage (post-treatment tumor maximum diameter: 6.6 cm). (**E**) Contrast-enhanced CT at 3 months post-surgery shows no evidence of residual or recurrent disease. (**F**) Histopathological findings: (Liver segments 6-7 tumor): Extensive sampling reveals ​extensive hemorrhagic necrosis, ​fibrosis, ​inflammatory cell infiltration, ​histiocytic reaction, and ​multinucleated giant cell reaction, consistent with post-therapeutic changes


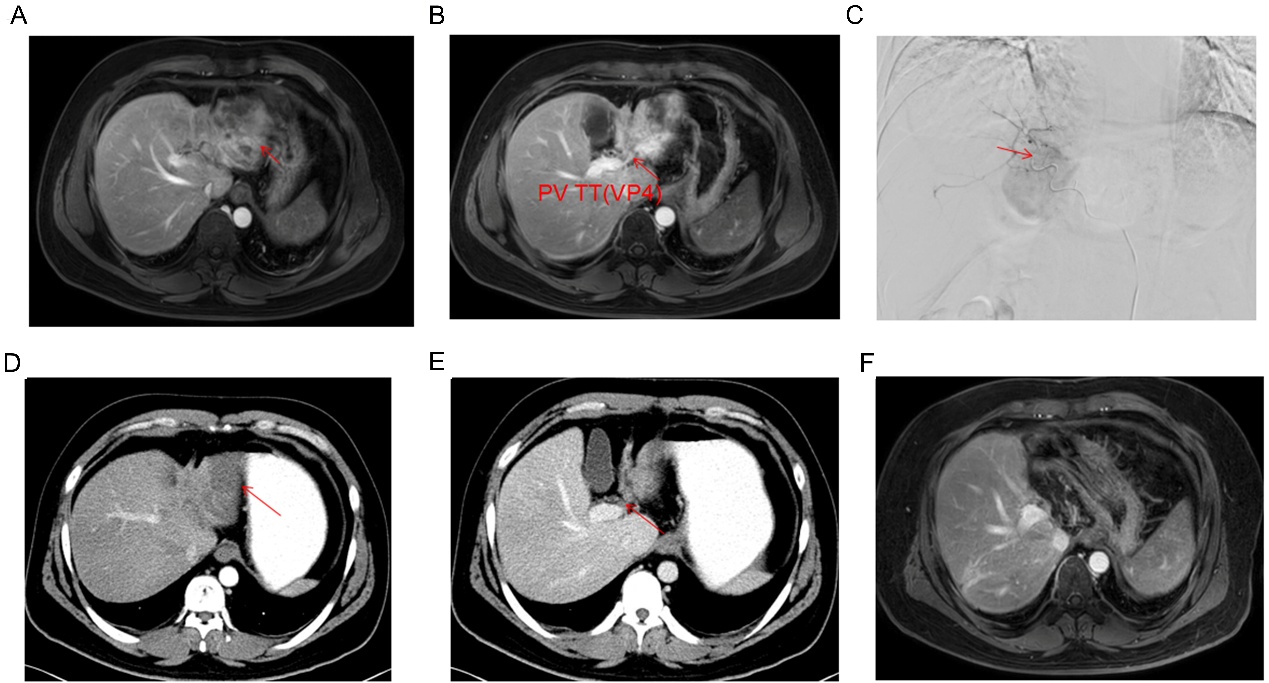


**Supplementary Figures S10.**

**Case 6.** (**A**) Pre-treatment imaging: Axial contrast-enhanced MRI (venous phase) demonstrates a large HCC lesion in the left-lobe (pre-treatment tumor maximum diameter: 10.1cm) and Vp4 PVTT (**B** red arrow). (**C**) Selective left hepatic arteriography confirms tumor staining and microcatheter tip positioning (red arrow) for HAIC. (**D**) Post-conversion therapy imaging: Follow-up Contrast-enhanced CT demonstrates significant tumor (red arrow) shrinkage (post-treatment tumor maximum diameter: 6.5cm) and regression of PVTT to segmental branches (**E** red arrow). (**F**) Contrast-enhanced CT at 3 months post-surgery shows no evidence of residual or recurrent disease.


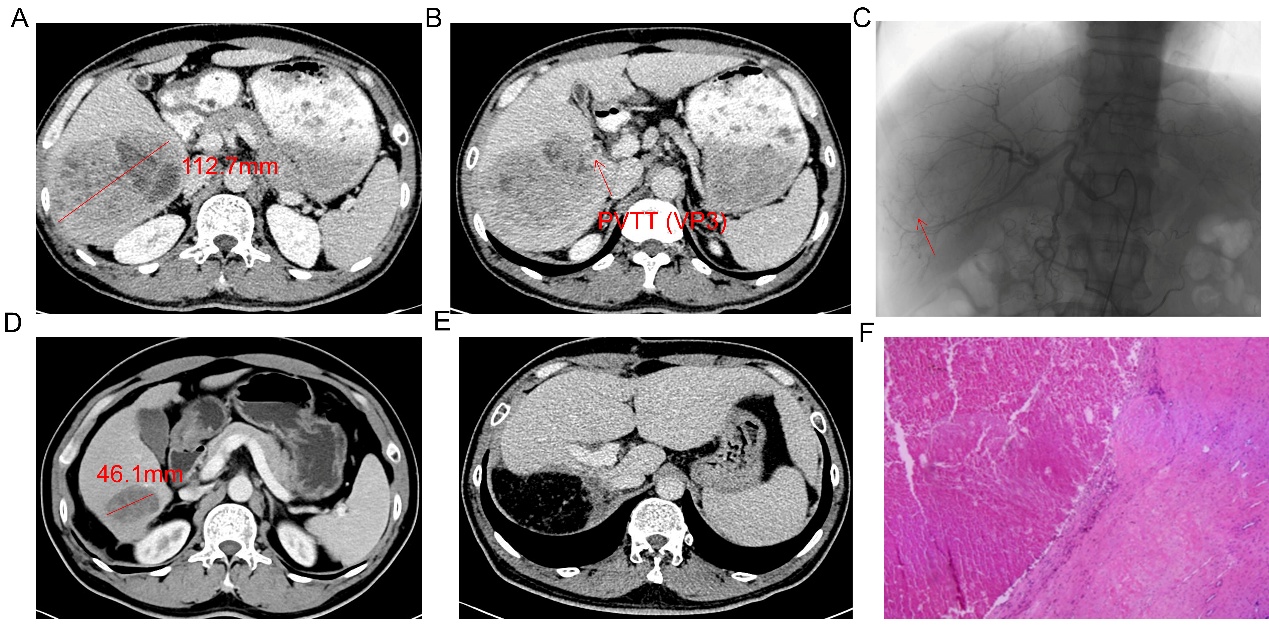


**Supplementary Figures S11.**

**Case 7.** (**A**)Pre-treatment imaging: Axial contrast-enhanced CT (venous phase) demonstrates a large HCC lesion in the right-lobe (pre-treatment tumor maximum diameter: 11.3cm) and Vp3 PVTT (**B** red arrow). (**C**) Selective hepatic arteriography demonstrates tumor staining (red arrow). (**D**) Post-conversion therapy imaging: Follow-up Contrast-enhanced CT demonstrates significant tumor shrinkage (post-treatment tumor maximum diameter: 4.6 cm). (**E**) Contrast-enhanced CT at 4 months post-surgery shows no evidence of residual or recurrent disease. (**F**)Histopathological findings: Treatment Efficacy: Complete Response (CR); ​Total Tumor Necrosis (No viable tumor cells identified); Quantitative Necrosis Assessment: Necrotic proportion: 50%; Residual viable tumor cells: 0%; Stromal component: 50%.


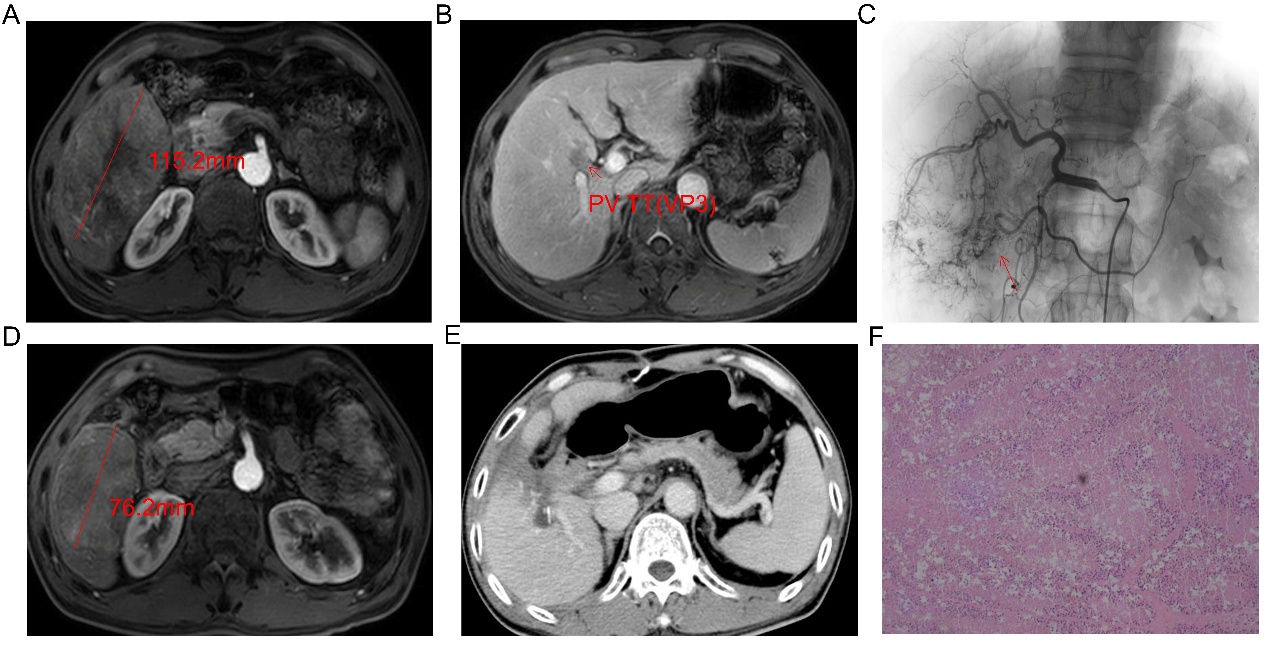


**Supplementary Figures S12.**

**Case 8.** (**A**)Pre-treatment imaging: Axial contrast-enhanced MRI (venous phase) demonstrates a large HCC lesion in the right-lobe (pre-treatment tumor maximum diameter: 11.5cm) and Vp3 PVTT (**B** red arrow). (**C**) Selective hepatic arteriography demonstrates tumor staining (red arrow). (**D**) Post-conversion therapy imaging: Follow-up Contrast-enhanced MRI demonstrates significant tumor shrinkage (post-treatment tumor maximum diameter: 7.6 cm). (**E**) Contrast-enhanced CT at 4months post-surgery shows no evidence of residual or recurrent disease. (**F**)Histopathological findings: Extensive sampling reveals ​extensive necrosis (80%) and ​degenerated tumor cells (10%), consistent with ​hepatocellular carcinoma (HCC) post-therapeutic changes. Stromal analysis: Stromal component: 10%, with ​cholesterol crystals observed within the stroma. Inflammatory infiltration, ​hemorrhage, ​histiocytic reaction, and ​multinucleated giant cell aggregates are present, further supporting treatment-related alterations.


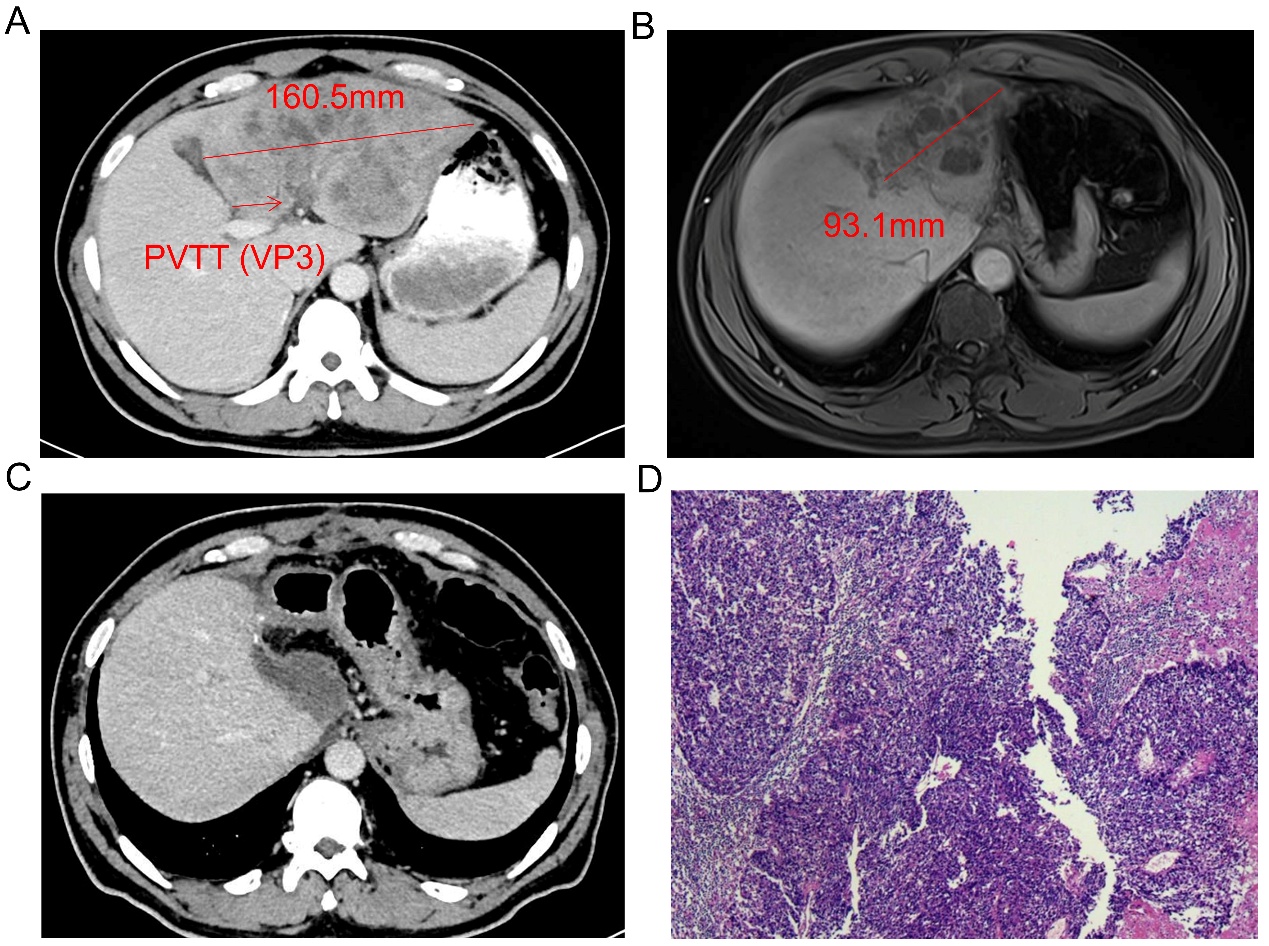


**Supplementary Figures S13.**

**Case 9.** (**A**) Pre-treatment imaging: Axial contrast-enhanced CT (venous phase) demonstrates a large HCC lesion in the Left-lobe (pre-treatment tumor maximum diameter: 16.1cm). (**B**) Post-conversion therapy imaging: Follow-up MRI reveals significant tumor shrinkage (residual diameter 9.3 cm). (**C**) Contrast-enhanced CT at 3 months post-surgery shows no evidence of residual or recurrent disease. (**D**)Histopathological findings: Incomplete Tumor Necrosis (viable tumor cells present); Quantitative Histopathological Assessment: Necrotic proportion: 80%Residual viable tumor cells: 10%; Stromal component: 10%.


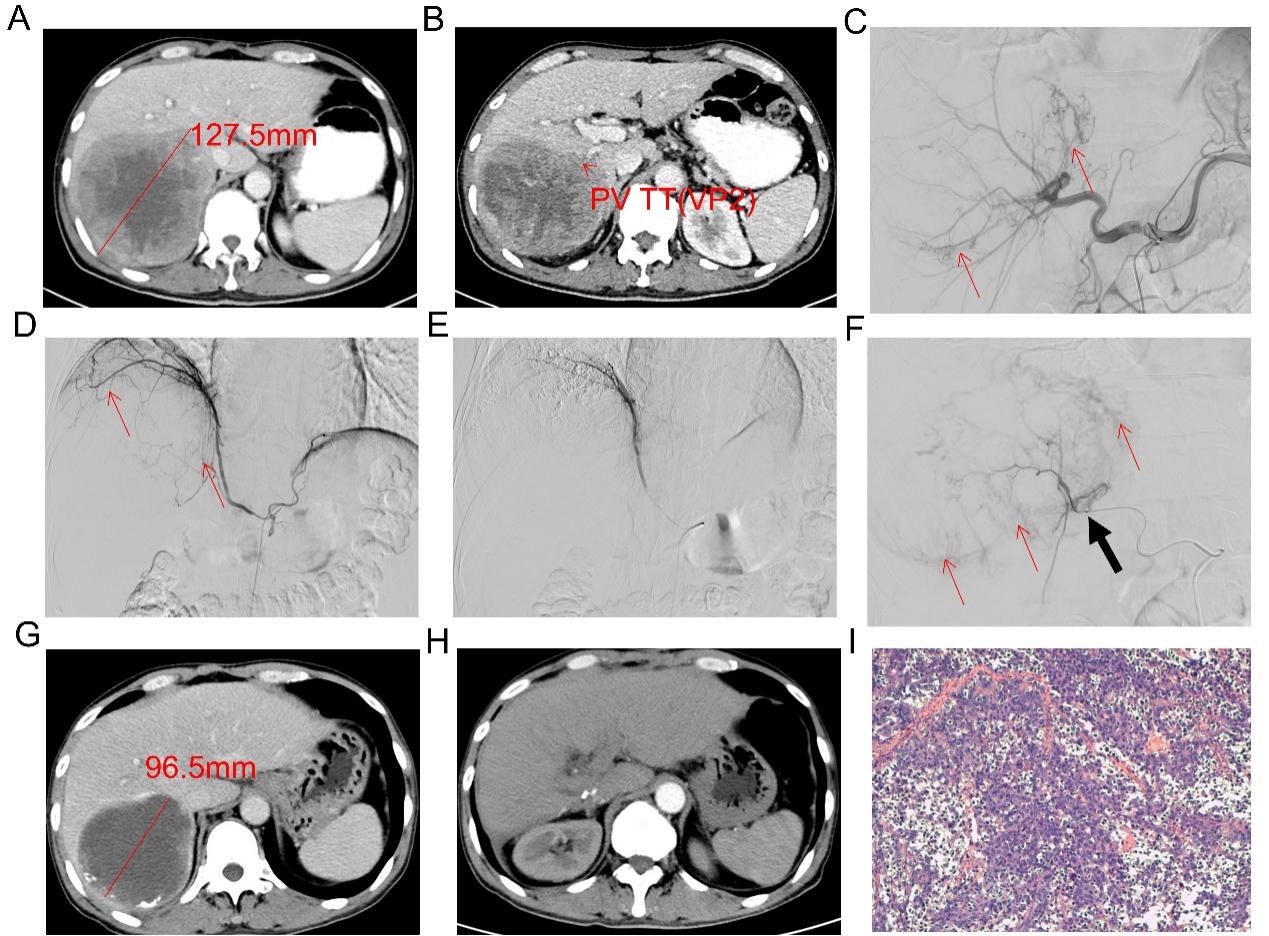


**Supplementary Figures S14.**

**Case 10.** (**A**) Pre-treatment imaging: Axial contrast-enhanced CT (venous phase) demonstrates a large HCC lesion in the right-lobe (pre-treatment tumor maximum diameter: 12.8cm) and Vp2 PVTT (**B** red arrow). (**C**)Selective hepatic arteriography demonstrates tumor staining (red arrow). (**D**)The catheter was advanced to the ​right inferior phrenic artery (RIPA) for angiography, revealing ​tumor staining in the right hepatic lobe (red arrow). (**E**) Subsequently, 20 mg of gelatin sponge particles (350–560 μm) were administered to embolize the RIPA. Post-embolization angiography confirmed ​complete disappearance of tumor staining. (**F**)Selective right hepatic arteriography confirms tumor staining (red arrow) and microcatheter tip positioning (black bold arrow) for HAIC. (**G**) Post-conversion therapy imaging: Follow-up Contrast-enhanced CT demonstrates significant tumor shrinkage (post-treatment tumor maximum diameter: 9.6 cm). (**H**) Contrast-enhanced CT at 3 months post-surgery shows no evidence of residual or recurrent disease. (**I**)Histopathological findings: Submitted liver tissue (left lateral lobe nodule) exhibits ​nodular cirrhosis with ​chronic inflammatory cell infiltration and ​localized fibrosis.


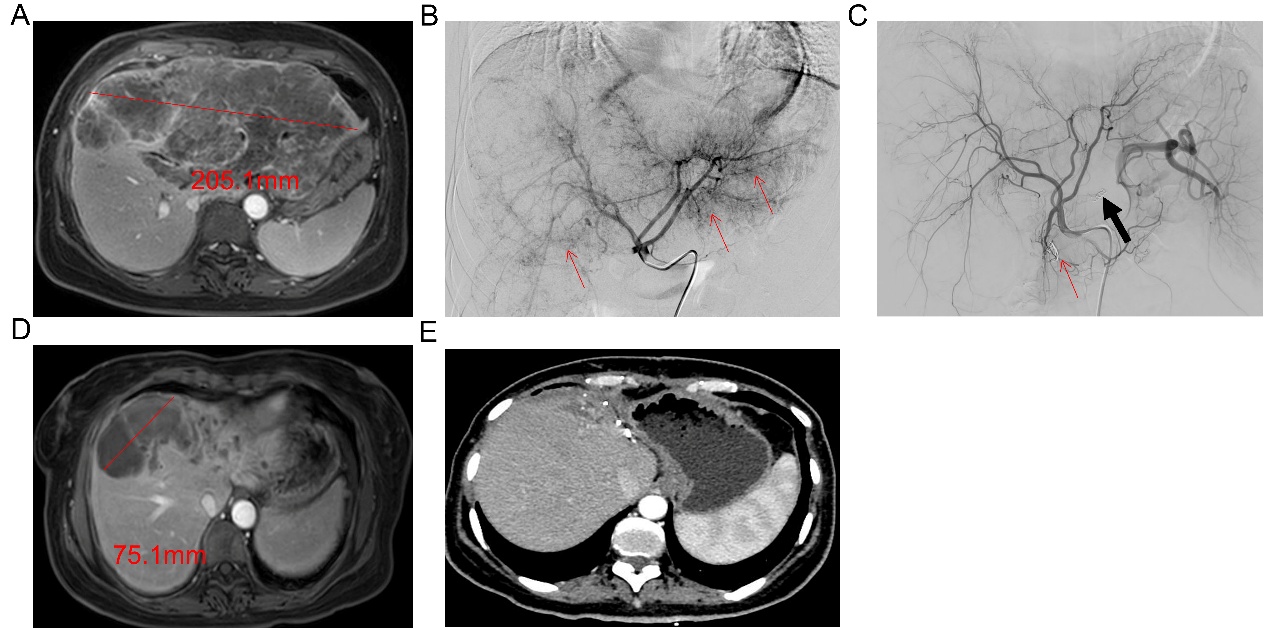


**Supplementary Figures S15.**

**Case 11.** (**A**) Pre-treatment imaging: Axial contrast-enhanced MRI (venous phase) demonstrates a large HCC lesion (pre-treatment tumor maximum diameter: 20.5cm).

(**B**)Selective hepatic arteriography demonstrates tumor staining (red arrow). (**C**)A embolization coil was used to perform ​protective embolization of the gastroduodenal artery (red arrow) and ​right gastric artery (black bold arrow)​. Subsequently, the microcatheter was retained in the ​proper hepatic artery for continuous ​chemotherapeutic agent infusion. (**D**) Post-conversion therapy imaging: Follow-up Contrast-enhanced MRI demonstrates significant tumor shrinkage (post-treatment tumor maximum diameter: 7.5 cm). (**E**) Contrast-enhanced CT at 5 months post-surgery shows no evidence of residual or recurrent disease.
