# Supplementary material for: Retrospective analysis of transarterial chemoembolization or hepatic arterial infusion chemotherapy combined with lenvatinib with or without PD-1 inhibitor as first-line therapy for unresectable hepatocellular carcinoma with high tumor burden: a propensity score-matched study
Source: Front Immunol. 2026 Feb 16;17:1717797. doi: 10.3389/fimmu.2026.1717797 (PMC12950717; doi:10.3389/fimmu.2026.1717797)
Supplement: Supplementary file 2 [file Table1.docx]

**Table S1 Primary Reasons for Non-Use of PD-1 Inhibitors in THL Cohort (n=139)**

| ​Category | ​Specific Reason | ​n (%)​ |
| --- | --- | --- |
| ​Financial Constraints | Excluded by national reimbursement policies | 77 (55.3%) |
|  | Out-of-pocket cost prohibitive | 18 (12.9%) |
| ​Patient Preference | Concerns about immune toxicity | 16 (11.5%) |
|  | Refusal of potential infusion reactions | 15 (10.7%) |
| ​Other | Concurrent COVID-19 infection | 5 (3.6%) |
|  | Unknown | 8(5.8%) |

**Abbreviations:** PD-1 inhibitors, programmed cell death protein 1 inhibilors; THL, Transarterial Chemoembolization Or Hepatic Arterial Infusion Chemotherapy combined with Lenvatinib
