# Supplementary material for: Retrospective analysis of transarterial chemoembolization or hepatic arterial infusion chemotherapy combined with lenvatinib with or without PD-1 inhibitor as first-line therapy for unresectable hepatocellular carcinoma with high tumor burden: a propensity score-matched study
Source: Front Immunol. 2026 Feb 16;17:1717797. doi: 10.3389/fimmu.2026.1717797 (PMC12950717; doi:10.3389/fimmu.2026.1717797)
Supplement: Supplementary file 3 [file Table2.docx]

**Table S2 Evidence-Based Selection Criteria for TACE vs. HAIC**

|  | ​TACE Preference | ​HAIC Preference |
| --- | --- | --- |
| ​ PVTT | Branch PVTT (Vp1–Vp3) | Main trunk PVTT (Vp4) |
|  | Partial main PVTT (<50% occlusion) | ≥50% luminal occlusion |
| ​Tumor Distribution | Unilobar disease (confined to 1 lobe) | Bilobar disease (≥1 lesion in each lobe) |
| ​Tumor Burden | ≤3 lesions | ≥4 lesions |
|  | Max diameter ≤10 cm | Diffuse infiltrative pattern |
| ​Liver Function Reserve | Child-Pugh A | Child-Pugh B7 |
|  | ALBI grade 1 | ALBI grade 2 |
| ​Technical Feasibility | Superselective catheterization achievable | Non-catheterizable lesions or arterial variants |

**Abbreviations:** TACE, transarterial chemoembolization; HAIC, hepatic arterial infusion chemotherapy; PVTT, portal Vein Tumor Thrombus
