# Supplementary material for: Retrospective analysis of transarterial chemoembolization or hepatic arterial infusion chemotherapy combined with lenvatinib with or without PD-1 inhibitor as first-line therapy for unresectable hepatocellular carcinoma with high tumor burden: a propensity score-matched study
Source: Front Immunol. 2026 Feb 16;17:1717797. doi: 10.3389/fimmu.2026.1717797 (PMC12950717; doi:10.3389/fimmu.2026.1717797)
Supplement: Supplementary file 4 [file Table3.docx]

Table S3 Subsequent treatments

| **Subsequent treatments** | **Number of patients** | |
| --- | --- | --- |
|  | **THL** | **THLP** |
| Apatinib | 0 | 5 |
| Apatinib + PD-1 inhibitors | 1 | 0 |
| Atezolizumab + bevacizumab | 2 | 1 |
| Iodine-125 seed implantation | 3 | 2 |
| Radiotherapy | 20 | 19 |
| Regorafenib | 0 | 23 |
| Regorafenib and PD-1 inhibitors | 18 | 1 |
| Regorafenib and PD-L1 inhibitors | 1 | 3 |
| Sintilimab + bevacizumab | 0 | 1 |

**Abbreviations:** PD-1 inhibitors, programmed cell death protein 1 inhibilors

PD-L1 inhibitors, Programmed Death-Ligand 1​ inhibilors

THL, Transarterial Chemoembolization Or Hepatic Arterial Infusion Chemotherapy combined with Lenvatinib;

THLP, Transarterial Chemoembolization Or Hepatic Arterial Infusion Chemotherapy combined with Lenvatinib and programmed death 1 inhibitors
