# Supplementary material for: Retrospective analysis of transarterial chemoembolization or hepatic arterial infusion chemotherapy combined with lenvatinib with or without PD-1 inhibitor as first-line therapy for unresectable hepatocellular carcinoma with high tumor burden: a propensity score-matched study
Source: Front Immunol. 2026 Feb 16;17:1717797. doi: 10.3389/fimmu.2026.1717797 (PMC12950717; doi:10.3389/fimmu.2026.1717797)
Supplement: Supplementary file 5 [file Table4.doc]

| **Patient** | **Treatment** | **SEX** | **Age** | **ECOG PS** | **AFP**  **(ng/ml)** | **HBsAg** | **CP Class** | **HTB Type** | **Largest tumor size** | **Tumor distribution** | **Number of tumors** | **Extrahepatic spread** | **Vascular invasion** | **BCLC stage** | **HAIC cycles** | **TTS** | **OS** |
| --- | --- | --- | --- | --- | --- | --- | --- | --- | --- | --- | --- | --- | --- | --- | --- | --- | --- |
| 1 | THL | Male | 51 | 1 | 4332 | Positive | A | Exceeded the up-to-11 criteria | 12.9 | Unilobar | >3 | No | PVTT (Vp3 ) | C | 2 | 3.5 | 33.7 |
| 2 | THLP | Female | 76 | 1 | 625 | Negative | B | VP4  PVTT | 4.5 | Unilobar | ≤3 | No | PVTT (Vp4 ) | C | 2 | 3.6 | 19.3 |
| 3 | THLP | Male | 53 | 1 | 287 | Positive | A | Exceeded the up-to-11 criteria | 10.3 | Unilobar | ≤3 | No | PVTT (VP3 ) | C | 3 | 4.1 | 44.7 |
| 4 | THLP | Male | 38 | 1 | 2650 | Positive | A | Exceeded the up-to-11 criteria | 12.7 | Unilobar | >3 | No | No | B | 4 | 3.6 | 18.6 |
| 5 | THLP | Male | 72 | 1 | 28462 | Positive | A | Exceeded the up-to-11 criteria | 12.8 | Bilobar | >3 | No | PVTT (Vp3 ) | C | 2 | 2.5 | 25.7 |
| 6 | THLP | Male | 36 | 0 | 1.79 | Positive | B | VP4  PVTT | 10.1 | Unilobar | ≤3 | No | PVTT (Vp4 ) | C | 4 | 4.7 | 28.8 |
| 7 | THLP | Male | 54 | 0 | 4853 | Negative | A | Exceeded the up-to-11 criteria | 11.3 | Unilobar | >3 | No | PVTT (Vp3 ) | C | 7 | 11.6 | 24.9 |
| 8 | THLP | Male | 53 | 0 | 15294 | Positive | A | Exceeded the up-to-11 criteria | 11.5 | Unilobar | ≤3 | No | PVTT (Vp3 ) | C | 2 | 2.1 | 27.4 |
| 9 | THLP | Male | 51 | 0 | 19100 | Positive | A | Exceeded the up-to-11 criteria | 16.1 | Unilobar | ≤3 | No | PVTT (Vp3 ) | C | 2 | 2.8 | 21.1 |
| 10 | THLP | Male | 56 | 1 | 60500 | Positive | A | Exceeded the up-to-11 criteria | 12.8 | Unilobar | ≤3 | No | PVTT (Vp2 ) | C | 2 | 3.2 | 22.9 |
| 11 | THLP | Female | 50 | 0 | 18243 | Positive | A | Exceeded the up-to-11 criteria | 20.5 | Bilobar | >3 | No | PVTT (Vp3 ) | C | 3 | 4.8 | 18.3 |

**Table S4 Baseline Characteristics and Imaging Features of Patients Achieving Conversion Resection**

**Abbreviations:** THL, Transarterial Chemoembolization Or Hepatic Arterial Infusion Chemotherapy combined with Lenvatinib; THLP, Transarterial Chemoembolization Or Hepatic Arterial Infusion Chemotherapy combined with Lenvatinib and programmed death 1 inhibitors

ECOG PS,**Eastern Cooperative Oncology Group** Performance Status; AFP, **Alpha-Fetoprotein**; CP Score,Child.Pugh.score;

HTB ,High tumor burden; HAIC,**Hepatic Arterial Infusion Chemotherapy**; TTS,Time interval from initial treatment to surgery;

OS,Overall survival;PVTT,Portal Vein Tumor Thrombus
