# Supplementary material for: Retrospective analysis of transarterial chemoembolization or hepatic arterial infusion chemotherapy combined with lenvatinib with or without PD-1 inhibitor as first-line therapy for unresectable hepatocellular carcinoma with high tumor burden: a propensity score-matched study
Source: Front Immunol. 2026 Feb 16;17:1717797. doi: 10.3389/fimmu.2026.1717797 (PMC12950717; doi:10.3389/fimmu.2026.1717797)
Supplement: Supplementary file 6 [file Table5.docx]

**Table S5 Longitudinal ALBI scores for both cohorts at all timepoints.**

| **Characteristic** | **Treatment** | | ***P*-value** |
| --- | --- | --- | --- |
|  | **THL  N = 139** | **THLP  N = 139** |  |
| **ALBI Score at baseline, Median (Q1, Q3)** | -2.50 (-2.79, -2.08) | -2.49 (-2.81, -2.06) | 0.751^1^ |
| **ALBI Score at 1month, Median (Q1, Q3)** | -2.26 (-2.64, -1.98) | -2.28 (-2.54, -1.95) | 0.688^1^ |
| **ALBI Score at 2month, Median (Q1, Q3)** | -2.37 (-2.68, -2.03) | -2.47 (-2.86, -2.03) | 0.176^1^ |
| **ALBI Score at 3month, Median (Q1, Q3)** | -2.29 (-2.72, -1.95) | -2.52 (-2.79, -2.09) | 0.102^1^ |
| **ALBI Score at 4month, Median (Q1, Q3)** | -2.24 (-2.65, -1.83) | -2.29 (-2.57, -1.88) | 0.660^1^ |
| **ALBI Score at 5month, Median (Q1, Q3)** | -2.30 (-2.66, -1.96) | -2.26 (-2.67, -1.82) | 0.345^1^ |
| **ALBI Score upon disease progression (Q1, Q3)** | -1.38 (-1.69, -1.02) | -1.35 (-1.71, -1.01) | 0.722^1^ |
| ^1^Wilcoxon rank sum test  ALBI, Albumin bilirubin;  THL, Transarterial Chemoembolization Or Hepatic Arterial Infusion Chemotherapy combined with Lenvatinib;  THLP, Transarterial Chemoembolization Or Hepatic Arterial Infusion Chemotherapy combined with Lenvatinib and programmed death 1 inhibitors | | | |
