# Supplementary material for: Retrospective analysis of transarterial chemoembolization or hepatic arterial infusion chemotherapy combined with lenvatinib with or without PD-1 inhibitor as first-line therapy for unresectable hepatocellular carcinoma with high tumor burden: a propensity score-matched study
Source: Front Immunol. 2026 Feb 16;17:1717797. doi: 10.3389/fimmu.2026.1717797 (PMC12950717; doi:10.3389/fimmu.2026.1717797)
Supplement: Supplementary file 7 [file Table6.docx]

**Table S6 Univariate and multivariate analysis of influencing factors associated with ORR in the all cohorts (Logistic regression)**

| **Characteristic** | **Univariable** | | | | | **Multivariable** | | | | |
| --- | --- | --- | --- | --- | --- | --- | --- | --- | --- | --- |
|  | **N** | **Event N** | **OR** | **95% CI** | **p-value^1^** | **N** | **Event N** | **OR** | **95% CI** | **p-value^1^** |
| **Treatment** |  |  |  |  |  |  |  |  |  |  |
| THL | 139 | 73 | — | — |  | 139 | 73 | — | — |  |
| THLP | 139 | 101 | 2.40 | 1.46, 3.96 | <0.001*** | 139 | 101 | 4.61 | 2.35, 9.04 | <0.001*** |
| **Trans-arterial therapies** |  |  |  |  |  |  |  |  |  |  |
| TACE | 100 | 59 | — | — |  | 100 | 59 | — | — |  |
| HAIC | 178 | 115 | 1.27 | 0.77, 2.10 | 0.354 | 178 | 115 | 1.38 | 0.69, 2.73 | 0.361 |
| **Etiology of Liver disease** |  |  |  |  |  |  |  |  |  |  |
| HBV | 228 | 141 | — | — |  | 228 | 141 | — | — |  |
| HCV | 50 | 33 | 1.20 | 0.63, 2.28 | 0.582 | 50 | 33 | 1.12 | 0.50, 2.51 | 0.789 |
| **Albumin bilirubin grade at screening** |  |  |  |  |  |  |  |  |  |  |
| 2 | 167 | 94 | — | — |  | 167 | 94 | — | — |  |
| 1 | 111 | 80 | 2.00 | 1.20, 3.35 | 0.008** | 111 | 80 | 1.70 | 0.83, 3.48 | 0.145 |
| **Child Pugh class at screening** |  |  |  |  |  |  |  |  |  |  |
| B | 64 | 33 | — | — |  | 64 | 33 | — | — |  |
| A | 214 | 141 | 1.81 | 1.03, 3.20 | 0.039* | 214 | 141 | 0.96 | 0.41, 2.23 | 0.924 |
| **High tumor burden type** |  |  |  |  |  |  |  |  |  |  |
| Exceeded the up-to-11 criteria And VP4 PVTT | 59 | 31 | — | — |  | 59 | 31 | — | — |  |
| VP4 PVTT | 76 | 51 | 1.84 | 0.91, 3.71 | 0.087 | 76 | 51 | 1.59 | 0.54, 4.74 | 0.401 |
| Exceeded the up-to-11 criteria | 143 | 92 | 1.63 | 0.88, 3.01 | 0.120 | 143 | 92 | 1.73 | 0.77, 3.93 | 0.187 |
| **Largest tumor size** |  |  |  |  |  |  |  |  |  |  |
| ˃11 | 132 | 73 | — | — |  | 132 | 73 | — | — |  |
| ≤11 | 146 | 101 | 1.81 | 1.11, 2.96 | 0.017* | 146 | 101 | 2.00 | 0.94, 4.27 | 0.074 |
| **Tumor distribution** |  |  |  |  |  |  |  |  |  |  |
| Bilobar | 164 | 94 | — | — |  | 164 | 94 | — | — |  |
| Unilobar | 114 | 80 | 1.75 | 1.06, 2.91 | 0.030* | 114 | 80 | 2.14 | 1.04, 4.40 | 0.039* |
| **Number of tumors** |  |  |  |  |  |  |  |  |  |  |
| ˃3 | 187 | 117 | — | — |  | 187 | 117 | — | — |  |
| ≤3 | 91 | 57 | 1.00 | 0.60, 1.68 | 0.991 | 91 | 57 | 0.71 | 0.33, 1.56 | 0.397 |
| **ΔALBI Score** |  |  |  |  |  |  |  |  |  |  |
| H | 139 | 88 | — | — |  | 139 | 88 | — | — |  |
| L | 139 | 86 | 0.94 | 0.58, 1.53 | 0.804 | 139 | 86 | 1.86 | 0.36, 9.72 | 0.463 |
| **ALBI Score change** |  |  |  |  |  |  |  |  |  |  |
| Worse | 154 | 100 | — | — |  | 154 | 100 | — | — |  |
| Better | 124 | 74 | 0.80 | 0.49, 1.30 | 0.368 | 124 | 74 | 0.37 | 0.07, 1.86 | 0.228 |
| **ALBI grade deterioration** |  |  |  |  |  |  |  |  |  |  |
| Yes | 72 | 46 | — | — |  | 72 | 46 | — | — |  |
| No | 206 | 128 | 0.93 | 0.53, 1.62 | 0.791 | 206 | 128 | 1.27 | 0.53, 3.00 | 0.593 |
| **Δ Child Pugh score** |  |  |  |  |  |  |  |  |  |  |
| H | 216 | 133 | — | — |  | 216 | 133 | — | — |  |
| L | 62 | 41 | 1.22 | 0.67, 2.20 | 0.514 | 62 | 41 | 0.24 | 0.08, 0.75 | 0.013* |
| **Child Pugh score change** |  |  |  |  |  |  |  |  |  |  |
| Worse | 99 | 56 | — | — |  | 99 | 56 | — | — |  |
| Stable | 117 | 77 | 1.48 | 0.85, 2.57 | 0.165 | 117 | 77 | 0.23 | 0.08, 0.66 | 0.006** |
| Better | 62 | 41 | 1.50 | 0.78, 2.90 | 0.229 | 62 | 41 |  |  |  |
| **Child Pugh class deterioration** |  |  |  |  |  |  |  |  |  |  |
| Yes | 91 | 27 | — | — |  | 91 | 27 | — | — |  |
| No | 187 | 147 | 8.71 | 4.93, 15.40 | <0.001*** | 187 | 147 | 31.75 | 11.42, 88.29 | <0.001*** |
| ^1^*p<0.05; **p<0.01; ***p<0.001 | | | | | | | | | | |
| Abbreviations: CI = Confidence Interval, OR = Odds Ratio | | | | | | | | | | |
| Null deviance = 368; Null df = 277; Log-likelihood = -127; AIC = 288; BIC = 350; Deviance = 254; Residual df = 261; No. Obs. = 278 | | | | | | | | | | |

THL, Transarterial Chemoembolization Or Hepatic Arterial Infusion Chemotherapy combined with Lenvatinib;

THLP, Transarterial Chemoembolization Or Hepatic Arterial Infusion Chemotherapy combined with Lenvatinib and programmed death 1 inhibitors

ALBI, Albumin bilirubin;
