# Supplementary material for: Retrospective analysis of transarterial chemoembolization or hepatic arterial infusion chemotherapy combined with lenvatinib with or without PD-1 inhibitor as first-line therapy for unresectable hepatocellular carcinoma with high tumor burden: a propensity score-matched study
Source: Front Immunol. 2026 Feb 16;17:1717797. doi: 10.3389/fimmu.2026.1717797 (PMC12950717; doi:10.3389/fimmu.2026.1717797)
Supplement: Supplementary file 8 [file Table7.docx]

Table S7 Cause of death in two groups.

| **Characteristic** | **Treatment** | | | **p-value** |
| --- | --- | --- | --- | --- |
|  | **Overall  N = 98** | **THL  N = 47** | **THLP  N = 51** |  |
| **Death of known, n (%)** |  |  |  | 0.423^1^ |
| Rrelated to liver failure | 92 (93.9%) | 43 (91.5%) | 49 (96.1%) |  |
| Unrelated to liver failure | 6 (6.1%) | 4 (8.5%) | 2 (3.9%) |  |
| **Specific Symptoms, n (%)**  **Rrelated to liver failure** |  |  |  |  |
| ​Hepatic encephalopathy | 13 (13.3%) | 10 (21.3%) | 3 (5.9%) |  |
| ​Hepatic encephalopathy and Jaundice | 1 (1.0%) | 0 (0.0%) | 1 (2.0%) |  |
| ​ Hepatic encephalopathy and Irreversible ascites and Variceal bleeding | 1 (1.0%) | 0 (0.0%) | 1 (2.0%) |  |
| Hepatorenal syndrome​ | 1 (1.0%) | 0 (0.0%) | 1 (2.0%) |  |
| Coagulopathy | 2 (2.0%) | 1 (2.1%) | 1 (2.0%) |  |
| Irreversible ascites | 35 (35.7%) | 16 (34.0%) | 19 (37.3%) |  |
| Irreversible ascites and coagulopathy | 2 (2.0%) | 0 (0.0%) | 2 (3.9%) |  |
| Irreversible ascites and Jaundice | 1 (1.0%) | 1 (2.1%) | 0 (0.0%) |  |
| Irreversible ascites and variceal bleeding | 1 (1.0%) | 1 (2.1%) | 0 (0.0%) |  |
| Irreversible ascites and Variceal bleeding | 2 (2.0%) | 2 (4.3%) | 0 (0.0%) |  |
| Jaundice | 16 (16.3%) | 6 (12.8%) | 10 (19.6%) |  |
| Variceal bleeding  **Unrelated to liver failure**  **Extrahepatic progression complications**   ​ Pulmonary embolism​  Ascites due to peritoneal carcinomatosis  Septic shock  **Other**  COVID-19 | 17 (17.3%)  1 (1.0%)  3 (3.1%)  1 (1.0%)  1 (1.0%) | 6 (12.8%)  0 (0.0%)  3 (6.4%)  1 (1.0%)  0 (0.0%) | 11 (21.6%)  1 (2.0%)  0 (0.0%)  0 (0.0%)  1 (2.0%) |  |
| ^1^Fisher's exact test | | | | |

**Abbreviations**: THL, Transarterial Chemoembolization Or Hepatic Arterial Infusion Chemotherapy combined with Lenvatinib;

THLP, Transarterial Chemoembolization Or Hepatic Arterial Infusion Chemotherapy combined with Lenvatinib and programmed death 1 inhibitors
