# Supplementary material for: Retrospective analysis of transarterial chemoembolization or hepatic arterial infusion chemotherapy combined with lenvatinib with or without PD-1 inhibitor as first-line therapy for unresectable hepatocellular carcinoma with high tumor burden: a propensity score-matched study
Source: Front Immunol. 2026 Feb 16;17:1717797. doi: 10.3389/fimmu.2026.1717797 (PMC12950717; doi:10.3389/fimmu.2026.1717797)
Supplement: Supplementary file 9 [file Table8.docx]

Table S8 Treatment-related adverse events

| **Adverse event** | **THLP, N = 139^1^** | |  |  | | **THL, N = 139^1^** | ***P*-value** | | |
| --- | --- | --- | --- | --- | --- | --- | --- | --- | --- |
|  | **Any Grade** | **Grade 3–4** |  | **Any Grade** | **Grade 3–4** | | **Any Grade** | | **Grade 3–4** |
| **Any adverse events** | 133 (95.7%) | 65 (46.8%) |  | 130 (93.5%) | 60 (43.2%) | | 0.597^3^ | | 0.629^2^ |
| **TACE or HAIC related AEs** | 37 (26.6%) | 8 (5.8%) |  | 44 (31.7%) | 6 (4.3%) | | 0.356^2^ | | 0.583^2^ |
| **Increased AST** | 65 (46.8%) | 8 (5.8%) |  | 42 (30.2%) | 4 (2.9%) | | 0.007^2^ | | 0.238^2^ |
| **Increased ALT** | 65 (46.8%) | 12 (8.6%) |  | 42 (30.2%) | 13 (9.4%) | | 0.007^2^ | | >0.999^2^ |
| **Elevated bilirubin** | 47 (33.8%) | 7 (5.0%) |  | 39 (28.1%) | 4 (2.9%) | | 0.299^2^ | | 0.356^2^ |
| **Thrombocytopenia** | 41 (29.5%) | 10 (7.2%) |  | 36 (25.9%) | 9 (6.5%) | | 0.503^2^ | | 0.812^2^ |
| **Elevated serum amylase** | 6 (4.3%) | 1 (0.7%) |  | 8 (5.8%) | 0 (0.0%) | | 0.583^2^ | | >0.999^3^ |
| **Decreased WBC count** | 41 (29.5% | 3 (2.2%) |  | 27 (19.4%) | 2 (1.4%) | | 0.051^2^ | | >0.999^3^ |
| **Neutropenia** | 25 (18.0%) | 4 (2.9%) |  | 16 (11.5%) | 1 (0.7%) | | 0.128^2^ | | >0.370^3^ |
| **Abdominal pain** | 46 (33.1%) | 2 (1.4%) |  | 31 (22.3%) | 1 (0.7%) | | 0.044^2^ | | >0.999^3^ |
| **Pyrexia** | 49 (35.3%) | 3 (2.2%) |  | 24 (17.3%) | 2 (1.4%) | | <0.001^2^ | | >0.999^3^ |
| **Hypertension** | 65 (46.8%) | 12 (8.6%) |  | 58 (41.7%) | 13 (9.4%) | | 0.398^2^ | | >0.999^2^ |
| **HFSR** | 40 (28.8%) | 5 (3.6%) |  | 28 (20.1%) | 4 (2.9%) | | 0.094^2^ | | >0.999^3^ |
| **Proteinuria** | 22 (15.8%) | 3 (2.2%) |  | 25 (18.0%) | 2 (1.4%) | | 0.631^2^ | | >0.999^3^ |
| **Fatigue** | 25 (18.0%) | 2 (1.4%) |  | 24 (17.3%) | 4 (2.9%) | | 0.875^2^ | | 0.684^3^ |
| **Vomiting** | 10 (7.2%) | 3 (2.2%) |  | 17 (12.2%) | 4 (2.9%) | | 0.156^2^ | | >0.999^3^ |
| **Nausea** | 10 (7.2%) | 1 (0.7%) |  | 18 (12.9%) | 1 (0.7%) | | 0.111^2^ | | >0.999^3^ |
| **Rash** | 17 (12.2%) | 4 (2.9%) |  | 20 (14.4%) | 1 (0.7%) | | 0.596^2^ | | 0.370^3^ |
| **Diarrhea** | 38 (27.3%) | 6 (4.3%) |  | 37 (26.6%) | 8 (5.8%) | | 0.893^2^ | | 0.583^2^ |
| **Decreased appetite** | 49 (35.3%) | 10 (7.2%) |  | 40 (28.8%) | 9 (6.5%) | | 0.247^2^ | 0.812^2^ | |
| **Weight loss**  **Immune-****related AEs**  **Any adverse events**  **Hypothyroidism**  **Hyperthyroidism**  **RCCEP**  **Pneumonitis**  **Hepatitis**  **Pruritus** | 45 (32.4%)  62 (44.6%)  58 (41.7%)  10 (7.2%)  8 (5.8%)  16 (11.5%)  9 (6.5%)  4 (2.9%) | 6 (4.3%)  7 (5%)  6 (4.3%)  0 (0.0%)  0 (0.0%)  1 (0.7%)  0 (0.0%)  0 (0.0%) |  | 38 (27.3%) | 4 (2.9%) | | 0.359  NA | 0.519^2^    NA | |

^1^n (%);

^2^Pearson's Chi-squared test;

^3^Fisher's exact test

**Abbreviations:**TACE, Transarterial Chemoembolization; HAIC, Hepatic Arterial Infusion, Chemotherapy; THL, TACE or HAIC combined with Lenvatinib;

THLP, TACE or HAIC combined with Lenvatinib and programmed death 1 inhibitors; AST, aspartate aminotransferase; ALT, alanine aminotransferase,

HFSR, hand-foot skin reaction; RCCEP reactive cutaneous capillary endothelial proliferation, WBC white blood cell count
